# Beyond Transgenic Mice: Emerging Models and Translational Strategies in Alzheimer’s Disease

**DOI:** 10.3390/ijms26125541

**Published:** 2025-06-10

**Authors:** Paula Alexandra Lopes, José L. Guil-Guerrero

**Affiliations:** 1 CIISA—Centro de Investigação Interdisciplinar em Sanidade Animal, Faculdade de Medicina Veterinária, Universidade de Lisboa, 1300-477 Lisboa, Portugal; ampalopes@fmv.ulisboa.pt; 2 Laboratório Associado para Ciência Animal e Veterinária (AL4AnimalS), Faculdade de Medicina Veterinária, Universidade de Lisboa, 1300-477 Lisboa, Portugal; 3 Departamento de Tecnología de Alimentos, Universidad de Almería, 04120 Almería, Spain

**Keywords:** Alzheimer’s disease models, neurodegeneration research, translational medicine, drug repurposing, predictive diagnostics, therapeutic strategies

## Abstract

Alzheimer’s disease (AD) is a leading cause of dementia and a growing public health concern worldwide. Despite decades of research, effective disease-modifying treatments remain elusive, partly due to limitations in current experimental models. The purpose of this review is to critically assess and compare existing murine and alternative models of AD to identify key strengths, limitations, and future directions for model development that can enhance translational relevance and therapeutic discovery. Traditional transgenic mouse models have advanced the understanding of amyloid-beta and tau pathologies, but often fail to capture the complexity of sporadic, late-onset AD. In response, alternative models—including zebrafish, *Drosophila melanogaster*, *Caenorhabditis elegans*, non-human primates, and human brain organoids—are gaining traction due to their complementary insights and diverse experimental advantages. This review also discusses innovations in genetic engineering, neuroimaging, computational modelling, and drug repurposing that are reshaping the landscape of AD research. By integrating these diverse approaches, the review advocates for a multi-model, multidisciplinary strategy to improve the predictive power, accelerate clinical translation, and inform personalised therapeutic interventions. Ethical considerations and equitable access to diagnostics and emerging treatments are also emphasised. Ultimately, this work aims to support the development of more accurate, effective, and human-relevant models to combat AD.

## 1. Introduction

### 1.1. Background on Alzheimer’s Disease

Alzheimer’s disease (AD) is a chronic neurodegenerative disorder and the leading cause of dementia, accounting for 60% to 70% of cases [1,2]. The condition is characterised by a progressive decline in memory, cognitive abilities, and behaviour, which ultimately results in impaired daily activities and social functioning [2,3]. While the disease typically manifests in individuals over 65 years of age, early-onset cases can occur before this threshold [1]. The precise aetiology of AD remains elusive, though it is believed to result from a combination of genetic and environmental factors [2,4]. Key pathological hallmarks of the disease include the accumulation of amyloid-beta (Aβ) plaques and neurofibrillary tangles composed of hyperphosphorylated tau protein, which contribute to neuronal dysfunction and cell death [2,5]. Furthermore, mitochondrial dysfunction and oxidative stress have been identified as crucial factors in AD progression, which further exacerbate neurodegeneration [2,5].

Ageing represents the most significant risk factor for AD, with incidence rates expected to rise as the global population continues to age [3,5]. Genetic predispositions, including mutations in the amyloid precursor protein (APP) and presenilin genes, as well as the presence of the apolipoprotein E4 allele, have also been linked to increased susceptibility [4]. Furthermore, environmental influences, such as traumatic brain injury (TBI) and vascular impairments, have been demonstrated to contribute to AD onset and progression [2,6]. The early detection of AD is imperative for effective disease management and the implementation of potential preventive measures. Cognitive screening tools, such as the Mini-Mental State Examination (MMSE) and clock-drawing tests, aid in identifying early cognitive impairment ([7]). In addition, the presence of biomarkers, including decreased Aβ-peptide and increased tau levels in cerebrospinal fluid, in combination with advanced imaging techniques such as MRI and PET scans, has been shown to improve the diagnostic accuracy [1,7].

As a major public health concern, AD currently affects approximately 44 million people worldwide, a number projected to double every 20 years [8]. The economic and social burdens of this disease are substantial, making it one of the leading causes of disability in old age [8,9]. AD remains a multifaceted disorder with profound implications for individuals and society. In fact, the impact extends beyond AD patients to caregivers, who often experience significant physical, emotional, and financial strain, underscoring the need for improved support systems and management strategies [8].

Research efforts are underway to elucidate the complex mechanisms underlying AD and to develop effective therapeutic interventions. Recent studies have exploited strategies targeting mitochondrial function and oxidative stress as potential treatment avenues [5]. Furthermore, the concept of “retrogenesis”, which suggests that AD progression mirrors brain development in reverse, provides new perspectives on disease management and care approaches [8].

Advancing research and improving early detection methods are critical for developing effective interventions and reducing the impact of AD. By integrating findings from genetic, environmental, and pathophysiological research, scientists continue working towards novel therapies aimed at slowing or preventing AD progression.

**Figure 1:**
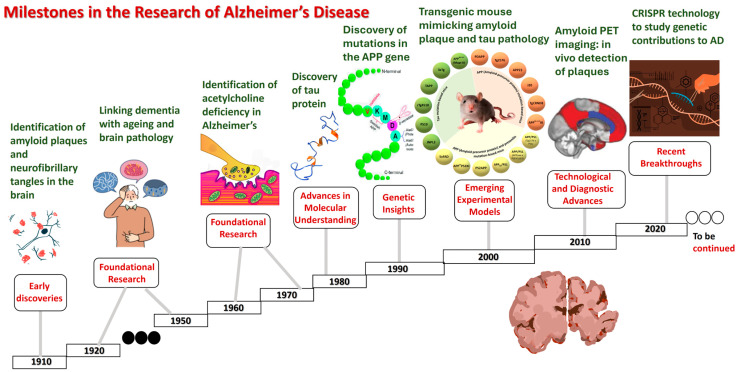
Milestones in Alzheimer’s disease research.

Milestones and the timeline in AD research are highlighted in Figure 1.

Early discoveries (1900s):

- 1906: Dr. Alois Alzheimer identifies the disease, describing amyloid plaques and neurofibrillary tangles in the brain of a patient.
- 1910: the term “Alzheimer’s disease” is coined by Emil Kraepelin.

Foundational research (1920s–1960s):

- 1920s–1930s: initial studies
- 1960s: the identification of an acetylcholine deficiency in Alzheimer’s brains sparks research into cholinergic therapies.

Advancements in molecular understanding (1980s):

- 1984: beta-amyloid protein is identified as a major component of plaques.
- 1986: the discovery of tau protein as a component of neurofibrillary tangles.
- 1987: the first AD drug, tacrine (Cognex), is approved for symptomatic treatment.

Genetic insights (1990s):

- 1991: the identification of mutations in the APP gene linked to early-onset AD.
- 1993: the apolipoprotein E (APOE) ε4 allele is identified as a risk factor for late-onset AD.
- 1997: presenilin genes (PSEN1 and PSEN2) are linked to familial AD.

Emerging experimental models (2000s):

- 2000s: the development of transgenic mouse models mimicking amyloid plaque and the tau pathology.
- 2003: the launch of the Alzheimer’s Disease Neuroimaging Initiative (ADNI) to identify biomarkers.
- 2004: the approval of memantine (Namenda), the first non-cholinergic AD drug.

Technological and diagnostic advances (2010s):

- 2012: the introduction of amyloid PET imaging, enabling the in vivo detection of plaques.
- 2013: genome-wide association studies (GWAS) identify new genetic risk factors.
- 2016: the identification of neuroinflammation as a significant contributor to AD progression.
- 2018: the approval of the tau PET tracer for studying tauopathy in living AD patients.

Recent breakthroughs (2020s):

- 2021: the FDA approves aducanumab (Aduhelm), the first amyloid-targeting drug, amid controversy over its efficacy.
- 2022: the emerging use of CRISPR technology to study genetic contributions to AD.
- 2023: lecanemab (Leqembi) gains FDA approval as a monoclonal antibody targeting amyloid plaques.
- Ongoing: advancements in human brain organoids and AI-driven diagnostics reshape research paradigms.

Future directions:

- Expanding research on late-onset AD and neuroinflammation.
- The development of non-invasive blood-based biomarkers for early detection.
- Exploration of lifestyle interventions and their impact on AD prevention.

### 1.2. Importance of Animal Models in AD Research

The use of animal models is of paramount importance in the realm of AD research for numerous reasons. Animal models provide vital insights into the complex pathogenesis of AD and enable researchers to dissect intricate mechanisms that are often inaccessible in human patients. For instance, studies using animal models have enhanced our understanding of AD by elucidating underlying genetic and molecular processes [10,11,12]. Transgenic models, such as the APP23 mouse, have been instrumental in uncovering the genetic basis of AD and the molecular pathology that underlies the disease [10,11].

Moreover, animal models are indispensable for testing novel therapeutic interventions. They provide a platform for the evaluation of the efficacy and safety of new drugs before their application in human clinical trials. Despite the limitations of current models in fully replicating the complexity of the human AD condition, they have yielded significant insights into disease progression and have contributed to the development of therapeutic strategies [13,14,15]. In this context, even the partial replication of the AD pathology by these animal models contributes to their predictive validity and underpins efforts to refine treatment approaches [11,13,14].

Robust animal models also mimic key disease features observed in AD. These models manifest histopathological and biochemical alterations, including amyloid plaques and tau tangles, that are pivotal for the study of disease progression and the evaluation of potential therapeutic interventions [10,11]. Furthermore, these models frequently replicate the cognitive and behavioural changes observed in AD patients, providing means to assess the impact of therapeutic interventions on memory and learning [10,16].

Despite their many advantages, animal models have inherent challenges and limitations. It is important to note that no single model can fully replicate the complex human condition of AD; each model represents only specific aspects of the disease [10,11]. Furthermore, there is a significant challenge in translating the findings from these models into successful human clinical outcomes, highlighting the need for the development of better models that integrate a broader range of genetic, environmental, and pathological factors [14,17].

Recent advancements in AD research have focused on the development of more comprehensive models that better mimic the sporadic form of the disease, which accounts for the majority of cases. For instance, novel models are being designed to combine genetic predispositions with environmental factors, such as diet and ageing [18]. Innovative techniques, such as optogenetics and chemogenetics, are being exploited in these models to elucidate the pathophysiological mechanisms of AD further and to pave the way for novel therapeutic strategies [19].

Notwithstanding their limitations, these animal models continue to serve as a cornerstone of preclinical research, guiding the design of human clinical trials and advancing our overall understanding of AD [10,11,13,14,17].

In summary, the use of animal models remains imperative in AD research, as they facilitate the acquisition of critical insights into the disease’s pathogenesis and the development of novel therapeutic interventions.

**Figure 2:**
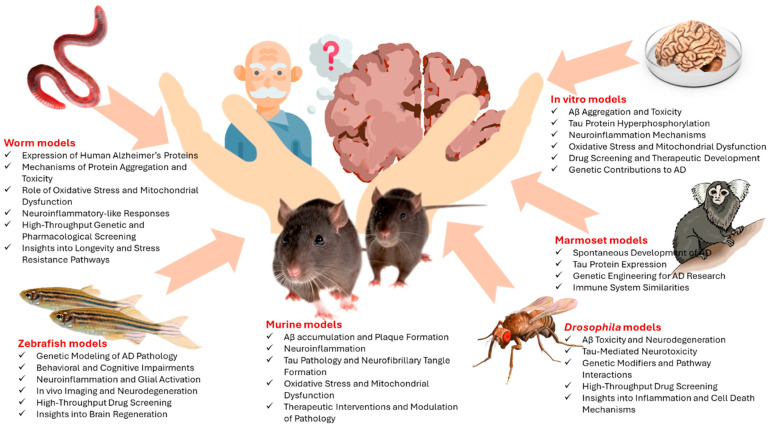
Key findings in AD research obtained from animal and in vitro models.

Key findings in AD research obtained from animal and in vitro models are detailed in Figure 2, and the timeline of models in AD research is given in Table 1.

## 2. Murine Models in AD Research

### 2.1. Development of Transgenic Murine Models for AD Research

Transgenic murine models have played a crucial role in advancing our understanding of AD and developing potential therapeutic interventions. These models are created by introducing familial AD-linked mutations into the mouse genome, leading to the expression of key pathological features, such as amyloid-β (Aβ) plaques, tau protein tangles, and neuronal loss [33,34,35,36,37]. By closely replicating aspects of human AD pathology, these models have become indispensable tools for studying disease progression and testing new treatments.

Murine models are commonly used to study these mutations, with some models combining multiple mutations to better replicate human disease pathology [38,39]. Despite their invaluable contributions to AD research, transgenic murine models have limitations. While they have provided crucial insights into disease pathophysiology, therapeutic testing, and genetic risk factors [33,40,41], they do not fully replicate human AD, particularly in terms of disease progression and neuronal loss. It is important to note that differences in the genetic background can also influence experimental outcomes, necessitating the careful interpretation of results and validation in human studies [34,42,43]. The most common transgenic murine models for AD research are detailed in Table 2.

A hallmark of transgenic models is their capacity to replicate the characteristic pathologies of AD. The majority of models manifest significant abnormalities, encompassing extracellular Aβ deposits, intracellular tau accumulation, and neuroinflammation, typified by microgliosis and astrogliosis [34,36,50]. Furthermore, behavioural deficits, such as memory loss and anxiety-like behaviours, have been observed, aligning with cognitive impairments seen in human AD patients [40,50]. Despite the challenges in replicating neuron loss, certain transgenic models have been observed to exhibit the significant degeneration of specific neuronal populations, including pyramidal and cholinergic neurons, which are particularly vulnerable in AD [36].

It is important to note that the complexity of the genetic makeup of these models can vary significantly. Single-gene models are predicated on the hypothesis that the expression of a single AD-related mutation, such as the PSEN1 V97L mutation found in Chinese familial AD [37], should be enhanced. More advanced multi-gene models, such as the 6xTg model, incorporate multiple mutations to better reflect the broad spectrum of AD pathology [50]. Second-generation models have been developed using humanised sequences and clinically relevant mutations in the endogenous mouse App gene, reducing the overexpression artefacts observed in earlier models [51].

The applications of transgenic murine models extend beyond basic research, offering valuable insights into the pathogenesis of AD. These models have been instrumental in studying mechanisms, such as the role of Aβ oligomers and tau pathology in neuronal dysfunction and cognitive decline [33,35,37]. Furthermore, they serve as essential platforms for the preclinical testing of potential AD treatments, including immunotherapies, small-molecule drugs, and nonviral DNA vaccines [33,41,52]. In addition, transgenic murine models have facilitated the identification of biomarkers, as longitudinal studies using magnetic resonance spectroscopy have detected metabolic changes that correlate with human AD biomarkers, supporting the development of diagnostic tools [52,53].

Despite their many advantages, transgenic murine models have limitations. It is notable that no single model fully recapitulates all aspects of human AD, particularly the early-stage pathologies and extensive neuronal loss that are characteristic of the disease [34,54]. Moreover, a significant challenge in AD research is the translational gap, wherein promising preclinical results in murine models fail to translate into effective treatments for human patients. This discrepancy underscores the necessity for more sophisticated and predictive models that more accurately reflect the complexity of human AD [54,55].

Although mouse models have traditionally dominated AD research due to their ease of genetic manipulation, short lifespan, and well-characterised genomes, they often fail to fully recapitulate the complex pathology and cognitive decline observed in human AD. In contrast, some rat models, such as the TgF344-AD rat, offer several advantages, including a larger brain size, more complex behaviour, and better translational relevance for cognitive testing [56]. This model carries human APP Swedish and PSEN1 ΔE9 mutations and develops a broad spectrum of AD-like pathology, including amyloid plaques, tau pathology, synaptic loss, gliosis, and age-related cognitive deficits, more closely mirroring human disease progression than many mouse models [57,58].

Overall, transgenic murine models remain invaluable tools in AD research, providing crucial insights into disease mechanisms and aiding the development of novel therapeutic strategies. Nevertheless, ongoing refinements are imperative to enhance their precision in modelling human AD and augment their relevance in translational research [33,34,36,37,40].

The development of future advances in the fields of genetic engineering and biomolecular techniques will be essential in establishing a connection between experimental findings in murine models and clinical outcomes in humans.

#### Key Genetic Modifications in Transgenic Murine Models for Alzheimer’s Disease

Transgenic murine models have played a pivotal role in the study of AD, particularly in the identification of genetic risk factors and the elucidation of pathological mechanisms. These models frequently incorporate mutations in genes linked to familial AD, resulting in the manifestation of hallmark features, such as amyloid plaques, neurofibrillary tangles, and cognitive deficits.

One of the most commonly used genetic modifications in AD research is the APPswe (Swedish mutation), which increases the production of Aβ peptides. This overproduction leads to the aggregation of Aβ into amyloid plaques, a defining characteristic of AD pathology [33,41,59]. In addition to the Swedish mutation, other mutations in the APP gene are used to study different aspects of amyloid pathology, thereby providing insight into variations in AD progression and response to therapies [60].

Beyond APP mutations, presenilin genes (PSEN1 and PSEN2) play a critical role in amyloid processing. Mutations in these genes, such as PSEN1 V97L, impact the γ-secretase complex, which is responsible for APP cleavage and Aβ peptide production [33,37,41]. In order to induce an AD-like pathology, researchers frequently use APP/PS1 double mutants, such as the APPswe/PS1dE9 model, which results in an increased amyloid plaque burden and cognitive impairment [60].

Another significant genetic element in the research field of AD pertains to tau protein mutations within the microtubule-associated protein tau (MAPT) gene. The tau pathology, typified by the formation of neurofibrillary tangles, constitutes a substantial component of AD and is associated with neuronal dysfunction and cell death. The utilisation of transgenic models incorporating MAPT mutations facilitates the investigation of the propagation of the tau pathology and its effect on brain function [33,37].

In addition to APP, PSEN, and tau mutations, the apolipoprotein E (apoE) gene is a crucial genetic factor in AD risk. The apoE4 allele, the strongest known genetic risk factor for late-onset AD, has been introduced into murine models to explore its role in amyloid deposition and neurodegeneration. Studies have shown that mice expressing human apoE4 exhibit increased Aβ aggregation and a disrupted lipid metabolism, contributing to neuronal damage and AD progression [41].

A comparison summarising the key characteristics of the specified mutations involved in familial AD (FAD) commonly used in murine models is detailed in Table 3. This Table provides a comparative overview of the familial Alzheimer’s disease mutations commonly used in animal models, highlighting their genetic basis, key features, and associated pathology. The age at which pathology becomes prominent varies among models. For example, the Swedish mutation in the Tg2576 mouse model shows early amyloid plaque formation [38,39]. These models are instrumental in elucidating disease mechanisms and evaluating potential therapeutic interventions, though it should be noted that they do not fully replicate the human AD condition [61,62].

In summary, transgenic murine models remain essential for AD research, particularly those involving APP, PSEN1/PSEN2, MAPT, and apoE mutations. While they have limitations in fully replicating human disease, they provide an essential framework for studying AD mechanisms and testing potential treatments. Continued advances in genetic engineering and biomolecular techniques will further refine these models, enhancing their relevance for translational research and drug development.

### 2.2. Knock-In and Injection Models in Alzheimer’s Research

AD research relies on a variety of animal models to investigate disease mechanisms and to evaluate potential therapies. Among these, knock-in and injection models are widely used, each offering distinct advantages and limitations. These models help researchers study different aspects of AD pathology, from amyloid plaque formation to cognitive decline, providing critical insights into disease progression and treatment strategies.

Knock-in models, such as AppNL-F to AppNL-G-F, offer a physiologically relevant approach by introducing familial AD mutations into the endogenous mouse APP gene. Unlike traditional transgenic models that overexpress amyloid precursor protein (APP), knock-in models maintain normal expression levels while still developing hallmark AD pathologies [67,68,69]. These models exhibit a significant amyloid plaque formation, neuroinflammation, and synaptic dysfunction, making them useful for studying the molecular underpinnings of AD [39,67,70]. Additionally, knock-in models develop age-dependent cognitive impairments, such as deficits in spatial memory and recognition tasks, allowing researchers to analyse disease progression and test therapeutic interventions [71,72,73].

Despite their advantages, knock-in models have certain limitations. While they effectively replicate the amyloid pathology, they often fail to develop a tau pathology or significant neuronal loss, which are also crucial aspects of AD [69]. Moreover, some models, such as AppNL-F, exhibit a delayed onset of symptoms, which can be a drawback when studying early-stage interventions [39].

Injection models provide an alternative approach by allowing for the targeted induction of AD pathology through the direct injection of the amyloid-beta peptide (Aβ1-42) into specific brain regions, such as the hippocampus [74,75]. This method has been shown to induce controlled pathological changes, making these models particularly valuable for short-term studies. Furthermore, injection models offer experimental versatility, as they enable researchers to test the effects of various peptides and compounds on AD pathology, facilitating drug discovery and therapeutic evaluations [74,76]. However, injection models also present notable challenges. Their invasiveness is a significant limitation, as intracerebroventricular (ICV) or intrahippocampal injections require direct surgical intervention, making them less suitable for studying the chronic and multifactorial nature of sporadic AD [76] (Ahn et al., 2020). Furthermore, these models do not fully replicate the progressive neurodegeneration observed in human AD, which may limit their applicability for long-term studies [74,75].

In conclusion, both knock-in and injection models are valuable tools in AD research, offering complementary insights into disease mechanisms. While knock-in models offer a more physiologically relevant representation of AD pathology, injection models allow for the precise experimental manipulation of key disease features. Despite their individual limitations, these models continue to drive advances in understanding AD and developing potential treatments. A comparison of knoci-In and injection models is exposed in Table 4.

### 2.3. Contributions of Murine Models for Understanding AD Pathogenesis

Murine models have significantly advanced our understanding of the pathogenesis of AD by providing crucial insights into pathological features, neuronal dysfunction, neuroinflammation, behavioural deficits, and therapeutic testing. Notable among these are the transgenic mouse models, such as APPswe/PS1dE9 and 3xTg-AD, which have been instrumental in the replication of hallmark features of AD, including Aβ plaques and neurofibrillary tangles [77,78]. These models have been instrumental in studying the progression of amyloid deposition and tau pathology, which are central to AD [78,79]. In addition, murine models have revealed an abnormal synaptic function, reorganisation, and altered neuronal firing activities that lead to excitation–inhibition imbalances [77,80]. Research has indicated that soluble Aβ oligomers are responsible for triggering synaptic dysfunction, while the presence of abnormal tau species has been linked to neuronal death and subsequent cognitive decline [80,81]. Furthermore, these models have emphasised the crucial role of neuroinflammation in the progression of AD. For instance, the J20 model has demonstrated that neuroinflammatory responses can precede Aβ deposition [82], and the modulation of the endocannabinoid system in the 5×FAD model has shown that enhancing the endocannabinoid tone can ameliorate memory deficits and reduce neuroinflammation [83]. The key findings of murine models for understanding AD pathogenesis is are detailed in Table 5. 

Behavioural assays, such as the Morris water maze and contextual fear conditioning, are vital for assessing cognitive functions and the impact of AD pathology on learning and memory [78,86]. The APP23 model, for instance, has been extensively applied to investigate cognitive impairments and behavioural alterations, thereby providing valuable insights into the disease’s effects on memory and anxiety [10,11]. Furthermore, mouse models are indispensable for the preclinical testing of potential therapeutic interventions; many treatments that have advanced to clinical trials were initially evaluated using these models [87,88]. Research has also demonstrated that targeting specific pathways, such as inhibiting Stearoyl CoA desaturase (SCD) or modulating the complement system, can restore cognitive functions and reduce AD pathology [85,89].

Overall, murine models have been indispensable in elucidating the complex mechanisms underlying AD. They provide critical insights into neuronal dysfunction, neuroinflammation, and cognitive impairments, and have been pivotal in testing potential therapeutic strategies. Despite their limitations, these models continue to serve as a cornerstone in AD research, effectively bridging the gap between basic science and clinical applications [78,79,80,81,86].

### 2.4. Limitations and Challenges of Murine Models

In order to address the limitations and challenges of murine models in AD research, it is essential to understand the various aspects in which these animal models fall short and the implications for translational research.

Murine models frequently fail to fully replicate the complexity of the human Alzheimer’s disease (AD) pathology, including the multifactorial nature of the disease and the heterogeneity of its symptoms [54,90,91,92]. The majority of these models are based on familial AD mutations, which represent only a small fraction of human cases, whereas the vast majority of AD cases are sporadic [91,93]. This fundamental difference limits their generalisability.

Moreover, significant anatomical and physiological differences exist between murine and human brains, complicating the translation of findings from animal models to clinical practice [54,91,94]. The progression and manifestation of AD in murine models often do not align with human disease, leading to discrepancies in therapeutic outcomes [54,95].

A major issue is the oversimplification of disease mechanisms. Many models are based primarily on the amyloid cascade hypothesis, which may not fully capture the complexity of AD [91,96]. This focus on amyloid-β pathology frequently overlooks other key pathological features such as the tau pathology, neuroinflammation, and synaptic dysfunction [63,68].

Encouraging preclinical outcomes in murine models do not consistently translate into successful clinical trials in humans, underscoring a major limitation in the predictive validity of these models [54,95,97]. This problem is exacerbated by the absence of reliable biomarkers and a lack of standardised endpoints in preclinical studies [95,97].

Additionally, murine models display a broad range of cognitive and behavioural impairments that may not accurately reflect the human AD condition [96,98]. The evaluation of these deficits and their correlation with neuropathological changes is often inconsistent [98,99].

Selecting the appropriate model and validating its relevance to human AD remains a challenge due to the wide diversity of available models and their specific characteristics [96,98]. Researchers must carefully choose models that best represent the particular aspects of AD they aim to investigate [98].

Recent advancements in genetic engineering and high-throughput technologies offer promising avenues for developing more predictive models, although their implementation remains in its early stages [95,100]. Integrating these innovations with cross-species comparisons could enhance the accuracy and translatability of the preclinical findings [94,100].

However, ethical concerns and practical limitations, such as the availability of human brain tissue for comparative studies, pose significant challenges [101]. Furthermore, longitudinal studies and comprehensive behavioural and pathological evaluations are needed, but these are resource-intensive [68,99].

The major limitations and challenges of murine models in AD research are detailed in Table 6.

In conclusion, while mouse models have significantly advanced our understanding of AD, their limitations and challenges necessitate careful consideration and innovative approaches to improve their translational relevance.

## 3. Emerging Alternative Models

### 3.1. Zebrafish Models

Zebrafish (*Danio rerio*) models have emerged as a valuable model for studying AD due to their genetic and physiological similarities to humans. These models provide important insights into the mechanisms of AD and offer advantages for high-throughput drug screening and genetic manipulation.

#### 3.1.1. Advantages of Zebrafish Models

Zebrafish possesses genes that are orthologous to those mutated in FAD, such as APP and presenilins, and these genes have conserved functions, making zebrafish a useful model for studying AD genetics [102,103,104]. Additionally, the basic brain structure and neurotransmitter systems in zebrafish, including cholinergic, glutamatergic, and GABAergic pathways, are highly conserved compared to mammals, which enables the study of neurodegenerative mechanisms [104,105].

Furthermore, zebrafish have been shown to be a valuable behavioural model for the assessment of cognitive and memory functions, which are critical for AD research [105,106]. This is due to the fact that zebrafish models possess behavioural assays that allow for the testing of learning, social interactions, and stress responses, thus making them an effective tool for evaluating disease progression. Moreover, the amenability of zebrafish to high-throughput drug screening is facilitated by their small size, rapid development, and transparent embryos, thereby enabling real-time imaging and analysis for drug discovery [105,107].

#### 3.1.2. Types of Zebrafish Models for AD

Transgenic zebrafish models have been developed to express human AD-related genes, such as APP and tau, thus allowing researchers to study disease-related molecular and cellular mechanisms [104,108]. These models facilitate the understanding of Aβ accumulation, tau hyperphosphorylation, and neuroinflammatory responses.

Pharmacological models of AD in zebrafish involve the use of chemicals, such as okadaic acid and aluminium chloride to induce an AD-like pathology. These models replicate key features of AD, including Aβ accumulation, tau hyperphosphorylation, and cognitive deficits, thus rendering them useful for studying disease mechanisms and therapeutic interventions [102,109,110,111]. Furthermore, zebrafish models have been employed to evaluate the efficacy of prospective therapeutic compounds, including lanthionine ketimine-5-ethyl esters and hydrogen-rich water, in mitigating AD pathology and enhancing behavioural outcomes [102,111].

#### 3.1.3. Research Findings

Gene function studies using zebrafish knockout models, such as abca7, have provided valuable insights into synaptic integrity, neurogenesis, and the molecular pathways involved in AD pathogenesis [108]. These findings help identify potential therapeutic targets for treating cognitive decline.

In addition, zebrafish models have been instrumental in the field of drug discovery, offering a rapid screening platform for the testing of new compounds and the understanding of their mechanisms of action [102,109,110]. The ability to visualise disease progression in real time has made zebrafish a crucial tool in the evaluation of the safety and efficacy of AD drug candidates.

#### 3.1.4. Challenges and Future Directions

Zebrafish models offer numerous advantages, yet further refinement and characterisation are needed to fully replicate the complexity of human AD [104,109]. A major limitation is the need to improve the translatability of zebrafish research findings to human disease models.

A more profound understanding of the neurophysiological properties of zebrafish is also necessary to enhance their utility in AD research. The application of advanced imaging techniques and electrophysiological assessments will facilitate the resolution of this discrepancy [112]. Future research should concentrate on integrating zebrafish models with other preclinical approaches, such as rodent models, to develop a more comprehensive understanding of AD.

In conclusion, zebrafish models offer a complementary approach to traditional rodent models in AD research, providing advantages in the domains of genetic manipulation, high-throughput screening, and real-time imaging. The continued development and characterisation of these models will enhance our understanding of AD and accelerate the discovery of effective treatment options.

### 3.2. Drosophila Models

*D. melanogaster*, commonly known as the fruit fly, has emerged as a powerful model organism for studying AD. This model offers several advantages, including the ease of genetic manipulation, a short lifespan, and the ability to produce large numbers of progeny, making it ideal for large-scale genetic screens and drug discovery [113,114,115,116].

#### 3.2.1. Key Features of Drosophila AD Models

*D. melanogaster* models allow for the overexpression of human genes associated with AD, such as the APP and beta-site APP cleaving enzyme 1 (BACE1), leading to the production of Aβ peptides [115,117]. These models exhibit key AD-related phenotypes, including amyloid plaque formation, neurofibrillary tangles, neuroanatomical changes, and behavioural deficits, such as impaired memory and motor functions [115,118,119,120]. Furthermore, AD phenotypes can be observed within a few days of the adult fly’s life, facilitating quick and efficient studies [115,118].

#### 3.2.2. Research Insights

Studies using the fruit fly, *D. melanogaster*, have yielded insights into the molecular functions of APP and its interactions, including the role of APP-like (APPL) in memory and brain plasticity [114,121]. These models have also facilitated the elucidation of the involvement of lipids and lipid signaling in AD pathology [122]. Furthermore, these models are used for the screening of potential therapeutic compounds, such as treatment with γ-secretase inhibitors, which have been shown to suppress AD phenotypes in these models [115]. In addition, compounds, such as thymoquinone, have demonstrated potential in reducing oxidative stress and improving behavioural outcomes in AD flies [123]. Research has also exploited the effects of nutraceuticals and interactions with the gut microbiome in ameliorating AD-associated phenotypes [114].

#### 3.2.3. Advantages of the Fruit Fly Model

Fundamental signalling pathways are highly conserved between fruit flies and mammals, making findings in flies relevant to human health [114]. The cost-effectiveness, ease of maintenance, and rapid life cycle of the fly make it a practical model for high-throughput genetic and drug screening [124].

In short, *D. melanogaster* serves as a valuable model for AD research, providing critical insights into disease mechanisms and facilitating the discovery of potential therapeutic agents. Its genetic tractability and the ability to recapitulate human disease phenotypes make it an indispensable tool on the ongoing battle against AD [113,114,115,118,124].

### 3.3. Worm Models

Worm models, particularly the nematode *C. elegans*, have been extensively used in the research field related to AD due to their simplicity, short lifespan, and well-characterised nervous system. These models facilitate the understanding of the molecular and cellular mechanisms underlying AD and in the screening of potential therapeutic compounds.

#### 3.3.1. Key Insights from Worm Models

One major insight from research on *C. elegans* relates to Aβ toxicity. The aggregation of Aβ peptides, a hallmark of AD, is effectively modelled in *C. elegans* [125]. For example, strains expressing human Aβ peptides, such as the CL2006 strain, exhibit paralysis and other neurodegenerative symptoms that are exacerbated by factors like copper exposure. These models facilitate not only a more profound understanding of the direct toxicity of Aβ, but also of how interactions with elements, such as metals, can further modulate this toxicity. In addition, behavioural and physiological studies have demonstrated that transgenic *C. elegans* expressing Aβ in specific neurons show altered responses, such as changes in CO_2_ sensitivity and deficits in chemotaxis and learning behaviour [126,127]. Such behavioural assays are crucial for establishing a link between molecular dysfunction and observable neurodegenerative symptoms. In addition, *C. elegans* models are extensively used for therapeutic screening. For instance, treatment with *Reineckia carnea* extract has been shown to reduce paralysis and neurotoxicity by inducing autophagy [128], while the use of a phospholipase D mutant has led to an improved motor performance and reduced neurodegeneration when Aβ is overexpressed [129]. Moreover, research into genetic and molecular mechanisms has revealed that pathways, such as the mitochondrial stress response and autophagy, play significant roles in maintaining cellular health and mitigating Aβ toxicity [130,131], providing valuable targets for enhancing cellular defence mechanisms. Finally, the integration of advanced technologies, such as the Wide Field-of-View Nematode Tracking Platform (WF-NTP), has enabled the high-throughput screening and detailed behavioural profiling of *C. elegans* models, thereby enhancing the sensitivity and reproducibility of large-scale studies on neurodegenerative diseases [132].

#### 3.3.2. Advantages of C. elegans in AD Research

*C. elegans* has a simple nervous system and is inexpensive to maintain, making it an ideal model for high-throughput genetic and drug screening. Many cellular pathways involved in proteostasis and stress responses are conserved between *C. elegans* and humans, providing relevant insights into human diseases [133]. Additionally, the short lifespan of *C. elegans* allows for the quick observation of disease progression and the effects of interventions.

In short, *C. elegans* models are invaluable tools in AD research, offering insights into the molecular mechanisms of the disease and enabling the screening of potential therapeutic compounds. Their simplicity, genetic tractability, and the development of advanced screening technologies make them a powerful system for studying neurodegeneration and testing new potential treatments [126,127,131,133].

### 3.4. Marmoset Models

Marmosets (*Callithrix jacchus*) are increasingly recognised as valuable models for AD research due to their physiological and pathological similarities to humans.

#### 3.4.1. Key Advantages of Marmosets in AD Research

❖ Primate-specific mechanisms: marmosets exhibit a greater degree of genetic, molecular, and cellular similarity with humans compared to rodents, rendering them more suitable for investigating primate-specific mechanisms underlying AD [134,135].
❖ The natural occurrence of AD pathologies: marmosets naturally develop key AD pathologies, such as Aβ plaques and tau protein abnormalities, which are critical for studying disease progression and potential treatments [135,136,137].
❖ A short lifespan: the shorter lifespan of approximately 8 years to the onset of ageing allows for more feasible longitudinal studies compared to other non-human primates, facilitating the study of ageing and neurodegeneration over a shorter period [136,138].

#### 3.4.2. Research Findings and Developments

❖ Genetic models: The MARMO-AD consortium has successfully generated gene-edited marmosets carrying PSEN1 mutations, which are associated with AD. These models are characterised by genetic, molecular, functional, behavioural, cognitive, and pathological features throughout their lifespan [134].
❖ Biomarkers of neural degeneration: Key biomarkers, including the total tau (T-tau), glial fibrillary acidic protein (GFAP), neurofilament light chain (NfL), and ubiquitin C-terminal hydrolase-L1 (UCH-L1), have been identified in marmosets. These biomarkers increase with age and are useful for evaluating neural health and therapeutic interventions [139].
❖ Tau protein studies: marmosets express both 3R and 4R tau isoforms, similar to humans, and exhibit tau phosphorylation at residues associated with AD, making them valuable for studying tau-related pathologies [137].
❖ Neuroinflammation and amyloidopathy: research has demonstrated that neuroinflammation can exacerbate amyloid plaque formation in marmosets, underscoring the role of the immune system in AD and offering novel perspectives for disease-modifying approaches [140].

All characteristics of marmoset models for AD research are summarised in Table 7.

In summary, marmosets can be considered a promising model for research into AD. This is due to several factors. Firstly, they naturally develop AD-like pathologies. Secondly, they exhibit genetic similarities with humans. Thirdly, longitudinal studies are feasible. These models have the potential to bridge the gap between rodent studies and human clinical trials. They may help to accelerate the development of effective novel therapies for AD.

### 3.5. In Vitro Models in AD Research

These models have been instrumental in advancing our understanding of the AD pathology and in the screening of potential therapeutic agents.

#### 3.5.1. 2D Cell Cultures

Two-dimensional (2D) cell cultures are valued for their simplicity and scalability, allowing researchers to perform high-throughput screening for basic research and drug testing [141,142]. Historically, these models have significantly contributed to the understanding of AD mechanisms and pathology [141,143]. However, their limitations become evident when considering the lack of complexity; two-dimensional models fail to replicate the intricate tissue architecture and cellular interactions of the human brain [141,142,144]. Furthermore, these cultures do not fully capture the in vivo environment, which is crucial for the study of neurodegenerative processes [144,145].

#### 3.5.2. 3D Cell Cultures and Organoids

**Figure 3:**
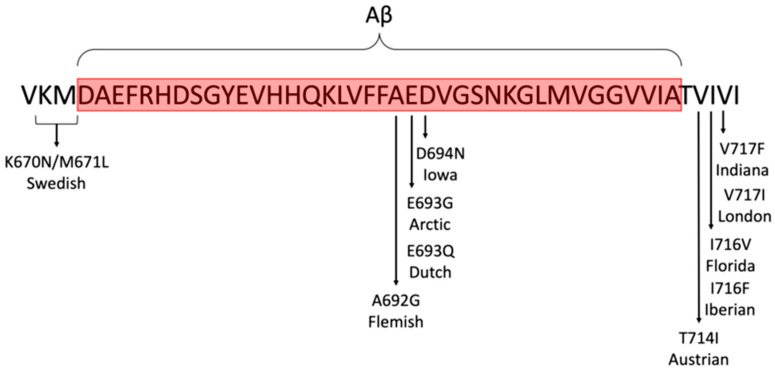
Familial AD mutations commonly used in animal models (file licensed under the Creative Commons Attribution-Share Alike 4.0 International license).

Three-dimensional (3D) cell cultures and organoids offer enhanced physiological relevance by better mimicking the brain’s structure and function. They incorporate multiple cell types, including neurons, astrocytes, and microglia, which facilitate complex cell–cell and cell–matrix interactions, thereby providing a more accurate representation of AD pathology [141,142,146,147]. Moreover, 3D brain organoids (Figure 3) are capable of recapitulating key features of AD, including amyloid plaques and neurofibrillary tangles, which render them particularly useful for disease modelling and drug testing [147,148,149]. However, 3D models are more complex and costlier to develop and maintain than 2D cultures [150,151]. Furthermore, despite their enhanced physiological relevance, 3D models still face challenges in fully replicating the complexity of the human brain, including aspects of ageing and regional brain differences [147,152].

#### 3.5.3. Other In Vitro Models

**Figure 4:**
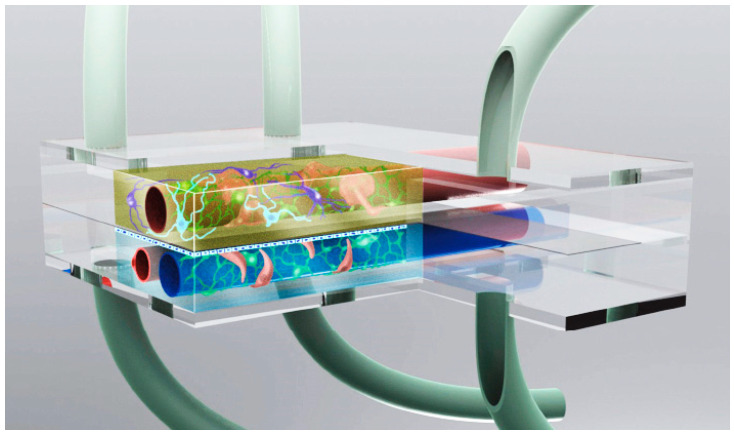
Neuronal microphysiological system display (image from the National Institute of Health).

Alternatively, in vitro approaches encompass co-culture systems and microfluidic devices. Co-culture systems, which combine different cell types, provide valuable insights into cell–cell interactions and the roles of glial cells in AD, offering a balance between simplicity and enhanced physiological relevance [151]. Microfluidic devices, also known as “organ-on-a-chip” systems (Figure 4), further extend the capabilities of in vitro models by integrating multiple tissue types and enabling a real-time analysis, thus offering a dynamic and versatile platform for AD research [153,154].

In summary, while 2D cell cultures offer simplicity and high throughput, 3D models and organoids provide a more physiologically relevant environment for studying AD. Other models, such as co-cultures and “organ-on-a-chip” systems, further contribute to this advance by enabling the study of complex cell interactions and dynamic responses. Collectively, these in vitro models enhance our understanding of AD and aid in the discovery of effective therapeutic interventions. A summary on In vitro models for AD research is exposed in Table 8.

### 3.6. Advantages and Disadvantages of Alternative Models for AD Research

As demonstrated in the preceding analysis of the various models, each one offers a unique perspective on AD. However, none of them fully captures the intricacies of the human AD condition. The integration of multiple models through a multidisciplinary approach, encompassing genetics, cellular biology, and behavioural sciences, serves to enhance the translational research. This, in turn, leads to the development of more effective therapeutic interventions. The subsequent Table 9 provides a synopsis of the salient points.

## 4. Technological Innovations in AD Model Research

### 4.1. Advanced Imaging Techniques

Advanced imaging techniques, such as Phase-Contrast X-Ray Imaging (XPCT) and other X-ray imaging methods, have been applied to animal models for AD research. Table 10 summarises the key features and applications of these advanced imaging techniques.

#### 4.1.1. X-Ray Phase Contrast Tomography (XPCT)

This technique offers exceptional spatial and contrast resolution, allowing for the detailed visualisation of amyloid plaques and their interactions with the neurovascular environment in AD mouse models. It preserves the tissue chemistry and structure, making it valuable for comparing physiological and pathological states [162].

#### 4.1.2. Phase-Contrast X-Ray-Computed Tomography (PCXCT)

PCXCT is highly sensitive and non-invasive, capable of detecting and quantifying high-density amyloid plaques without the need for imaging agents [163]. It provides detailed 3D visualisation and reveals age-related changes in the plaque density.

#### 4.1.3. Diffraction Enhanced Imaging (DEI)

DEI offers a higher soft tissue contrast and higher resolution than conventional MRI, making it effective for visualising small amyloid plaques and anatomical structures in AD mouse brains [162]. While showing promise for early diagnosis, this technique is still in the proof-of-principle stage [164].

#### 4.1.4. Analyser-Based X-Ray Imaging (ABI)

ABI provides high contrast and spatial resolution using monochromatic X-rays. It has potential for in vivo applications and offers a detailed depiction of anatomical structures, although it faces technical challenges in live animal imaging [165].

The employment of these advanced imaging techniques has been demonstrated to enhance the ability to study AD in animal models, providing detailed insights into amyloid plaque formation and progression. Furthermore, these imaging techniques offer the potential for the early diagnosis and monitoring of therapeutic interventions.

### 4.2. Computational Approaches and Machine Learning in AD Model Analysis

The application of machine learning (ML) approaches in AD research using animal models has demonstrated substantial advances in predicting disease onset, elucidating underlying mechanisms, and assessing potential treatments. Through the integration of neuroimaging, genetic, behavioural, and biochemical data, ML techniques provide novel insights that augment the translational value of animal studies for human AD research (Table 11).

#### 4.2.1. Supervised Learning

Techniques such as CatBoost, SVM, and Decision Trees have demonstrated a high degree of accuracy in predicting AD using clinical and MRI data [166].

#### 4.2.2. Neuroimaging Analysis

Advanced deep learning models, notably VGG16, have exhibited a marked superiority over conventional ML algorithms in the analysis of MRI and PET scans [167].

#### 4.2.3. Genetic Data Analysis

The performance of ML algorithms varied (Area Under Curve (AUC) 0.59–0.98), with potential biases in feature selection and validation [168].

#### 4.2.4. Multi-Modal Analysis

The combination of clinical, genetic, and neuroimaging data enhanced predictive capabilities, emphasising the importance of diverse biomarkers [169,170].

#### 4.2.5. Ensemble Methods

Techniques such as Random Forests, XGBoost, and Voting Classifiers have been shown to achieve high accuracy, thereby demonstrating the robustness of ensemble methods [171].

#### 4.2.6. Hybrid Models

The combination of multiple ML algorithms has been demonstrated to enhance the prediction accuracy, with hybrid models achieving up to 95.12% accuracy [172].

#### 4.2.7. Deep Learning

Transfer learning models like VGG16 show high potential in enhancing the diagnostic accuracy for AD [167].

#### 4.2.8. Non-Invasive Techniques

ANN models using cognitive test data provide high F1-Scores, indicating their effectiveness in early AD detection [173].

These insights highlight the diverse computational approaches and the potential of ML in advancing AD research, diagnosis, and treatment. ML has revolutionised AD research using animal models, offering high-precision diagnostic tools, genetic insights, and optimised drug discovery pipelines. However, challenges in the translational validity, bias reduction, and computational complexity must be addressed to fully leverage ML’s potential in AD research. It is anticipated that advancements in hybrid models, multimodal integration, and explainable artificial intelligence (AI) will lead to the enhanced applicability of ML-driven discoveries from animal studies to human clinical trials. This, in turn, is expected to result in improvements in early AD detection and treatment options.

### 4.3. Omics Technologies for Biomarker Discovery

Omics technologies have been demonstrated to play a crucial role in identifying biomarkers for AD by providing a comprehensive understanding of metabolic, proteomic, transcriptomic, and genomic alterations [174]. These omics approaches enhance the diagnostic accuracy, facilitate drug evaluation, and uncover disease mechanisms using both human and animal models [175].

Metabolomics, for instance, utilises techniques such as ultra-performance liquid chromatography coupled with quadrupole time-of-flight tandem mass spectrometry (UPLC-Q-TOF-MS) to analyse metabolic changes in plasma, hippocampus, and cortex samples of AD rat models. This approach has identified potential biomarkers, such as lysophosphatidylcholine (LysoPC) and intermediates of sphingolipid metabolism [176]. Conversely, the field of proteomics utilises high-throughput analysis techniques, such as liquid chromatography–mass spectrometry (LC-MS) and other mass spectrometry-based methods, to detect protein biomarkers in cerebrospinal fluid (CSF) and plasma [177,178].

Metabolomic analysis in AD rat models, for instance, has been employed to identify biomarkers that differentiate between diseased and healthy states, facilitating early AD diagnosis [176,179]. Furthermore, these biomarkers aid towards the assessment of the efficacy of therapeutic interventions, such as the effects of donepezil and pine nuts on AD biomarkers in rat models [176]. Animal models also enable researchers to study metabolic networks and pathways involved in AD, which can be later translated into human studies [179]. ML integration further enhances the potential of omics data by improving data analysis and biomarker discovery. ML techniques process large-scale omics datasets, integrate information from different sources, and model disease heterogeneity [180,181]. Automated ML tools like JADBIO have been successfully used to construct predictive models for AD diagnosis based on omics data, demonstrating the potential for minimally invasive blood-based diagnostic tests [182].

Transcriptomics facilitates the study of alterations in gene expression through techniques such as RNA sequencing and real-time quantitative reverse transcription PCR (qRT-PCR), enabling the identification of mRNA and microRNA biomarkers [177,183]. Concurrently, genomics employs DNA sequencing and single-nucleotide polymorphism (SNP) analysis to unravel genetic risk factors and biomarkers associated with AD [183].

In the context of animal models, omics technologies offer a range of applications, including disease diagnosis, drug evaluation, and mechanistic studies (Table 12). By leveraging these advanced technologies, researchers continue to refine biomarker identification, improve early AD diagnosis, and develop more effective novel treatment strategies.

In summary, it is evident that omics technologies, encompassing metabolomics, proteomics, transcriptomics, and genomics, play a pivotal role in the identification of biomarkers for AD through the use of animal models. These omics technologies not only facilitate early diagnosis and drug evaluation, but also offer insights into the molecular mechanisms underlying AD. The integration of omics data with machine learning further enhances the potential for developing predictive models and for personalising therapeutic strategies [176,177,179,180,182].

## 5. Comparative Analysis of Mouse and Alternative Models

### 5.1. Translational Discrepancies Between Models

The field of AD research is heavily reliant on the use of animal models to facilitate a comprehensive understanding of the underlying disease mechanisms and to evaluate the efficacy of potential therapeutic interventions. However, a conspicuous disparity exists between the findings observed in these models and the complex pathological characteristics of human AD [54]. This incongruity has the potential to delay the development of effective therapeutic strategies. It is, therefore, imperative to comprehensively appraise the strengths and limitations of diverse models in order to enhance their relevance to human AD and to advance therapeutic interventions.

#### 5.1.1. Mouse Models in AD Research

Transgenic mouse models, which express the human amyloid precursor protein (hAPP) and Aβ, are extensively used to study AD. These animal models have been instrumental in understanding the disease pathogenesis and testing potential therapeutic interventions [96,184,185]. However, traditional transgenic models frequently exhibit expression artefacts, resulting in physiological differences that may not fully replicate human AD [184].

Recent advancements have led to the development of knock-in models, such as AppNL−G−F, which circumvent these expression issues while still demonstrating a significant amyloid plaque burden [67]. Nonetheless, these models may not exhibit robust cognitive deficits, limiting their ability to fully model the disease’s cognitive impact [67].

In addition to genetic modifications, researchers have recognised the importance of the genetic background in influencing the disease pathology. Studies suggest that differences in the genetic background can significantly affect the brain proteome and AD-related pathways, emphasising the need for greater genetic diversity in model selection to improve the translational validity [186].

#### 5.1.2. Emerging Alternatives to Murine Models

Murine models have dominated research in AD, but there is an increasing exploration of alternative organisms to overcome their limitations. Small model organisms, such as *C. elegans*, *D. melanogaster*, and *Danio rerio* have been used to study amyloid-related processes. These models share transcriptomic similarities with mice in biological processes, such as protein misfolding and the immune response, making them valuable for high-throughput genetic and pharmacological screening [187].

Beyond small models, non-human primates, such as marmosets are gaining attention for their potential to bridge the gap between rodent studies and human AD [134]. With their closer genetic and physiological similarities to humans, marmosets offer a promising platform for studying primate-specific mechanisms underlying AD, potentially overcoming some limitations of rodent models. Other species, such as the degu and the dog, have also been considered promising due to their ability to better recapitulate human AD neuropathology and cognitive impairment [187].

#### 5.1.3. Translational Discrepancies

Despite their value, contemporary animal models frequently fail to fully replicate the complex aetiology and heterogeneous pathology of human AD [54,96,184]. Differences in early-stage pathologies and behavioural phenotypes between mice and humans highlight the limitations of rodent models in fully capturing AD progression [54,184]. Furthermore, comparative studies at the transcriptomic and proteomic levels reveal that while some molecular pathways are conserved, others differ significantly between species, affecting the reliability of translating findings from animal models to human clinical trials [188,189]. These translational discrepancies contribute to the high failure rate of AD therapies in human trials. The failure of numerous treatments that demonstrate efficacy in mouse models to translate to clinical trials underscores the necessity for models that replicate human AD more accurately [54,188]. Addressing these limitations is thus imperative for the development of more effective AD treatment options.

#### 5.1.4. Improved Translation Recommendations

It is recommended that greater genetic diversity be incorporated into mouse models in order to improve the translational value of AD research. This is due to the fact that greater genetic variability can help capture a broader range of molecular heterogeneity, making models more representative of human AD [186]. Additionally, it is essential for enhancing model reliability that comprehensive study designs, including rigorous behavioural assessments and biomarker use, be implemented [54].

Furthermore, the combination of diverse models, including non-human primates and alternative species, can facilitate a more comprehensive understanding of AD [134,187]. This multi-model approach enables researchers to integrate findings across different systems, reducing reliance on a single model and enhancing the overall translational pipeline. The comparison of translational discrepancies between mouse and alternative AD models is summarised in Table 13.

#### 5.1.5. Key Points

##### Mouse Models

These models are extensively applied due to their capacity to replicate salient pathologies associated with AD through genetic manipulation. Nevertheless, there is often a discrepancy between the preclinical findings and clinical outcomes, attributable to disparities in the AD progression and pathology between mice and humans [54,81,192].

##### Alternative Models

These include species, such as the degu, dog, and non-human primates, which have been shown to better replicate human AD pathology and cognitive impairment [187,188,193]. These alternative models are less reliant on genetic modifications and may offer higher translational potential. However, it should be noted that there are discrepancies between preclinical studies using mouse models and subsequent human trials. This is partly due to the artificial nature of these models and their inability to fully capture the complexity of human AD [54,81,192]. Conversely, alternative models have been shown to offer insights into the mechanisms of human AD due to their closer resemblance to human disease mechanisms [187,188].

##### Molecular and Behavioural Assessments

Both mouse and alternative models are employed in the study of molecular changes and cognitive deficits associated with AD. However, the selection of a model and the specific assessments employed can considerably influence the translational relevance of the findings [188,190,195].

In conclusion, while mouse models remain a cornerstone in AD research, alternative models offer promising avenues for improving the translational success of preclinical findings. It is, therefore essential for research advancement and therapeutic development in AD that a nuanced understanding of each model’s strengths and limitations is fully obtained.

### 5.2. Strengths and Weaknesses of Different Model Systems for Alzheimer’s Disease Research

The study of AD employs diverse model systems, each contributing valuable insights into disease mechanisms and therapeutic strategies. However, these models have significant limitations, necessitating a multimodal approach to improve the translational outcomes (Table 14). Transgenic mouse models have been instrumental in replicating key AD features—such as Aβ plaques, tau tangles, and synaptic deficits—and support genetic manipulation and longitudinal studies. Yet, they often fail to model sporadic AD, which represents over 90% of human cases, largely due to differences in aging and immune responses. This gap may partly explain why many treatments effective in mice do not succeed in clinical trials [18].

The 3xTg-AD mouse model combines amyloid and tau pathologies, allowing the study of their interaction. While this addresses a limitation of single-pathology models, its use of rare familial mutations and omission of environmental influences reduces its relevance to sporadic AD [23]. Rat models offer advantages in their brain size and cognitive capacity, enhancing behavioural and pharmacokinetic research. However, they often rely on artificial amyloid injections, limiting their ability to capture the progressive pathology [158].

Invertebrate models, such as *D. melanogaster* and *C. elegans*, are ideal for high-throughput genetic and drug screening due to their simplicity and low cost. These models have provided insights into molecular pathways, though their lack of complex brain structures limits their relevance to cellular and network-level AD pathology [24]. Non-human primates share high anatomical and physiological similarity with humans, making them valuable for studying ageing and amyloid pathology. However, ethical concerns, high costs, and slow disease progression restrict their application to later-stage preclinical research [196].

Human-derived models, including iPSC-derived neurons and brain organoids, facilitate patient-specific studies that more accurately reflect sporadic AD mechanisms. However, these models are constrained by a lack of cellular diversity, immature neuronal networks, and the absence of vasculature or immune components—factors that are essential for disease progression [25,26]. Innovative platforms such as the blood–brain barrier (BBB) on a chip provide controlled environments to investigate amyloid transport and drug delivery, but oversimplify the complex cellular interactions of the BBB [197].

Finally, CRISPR-engineered models allow for the precise introduction of familial AD mutations, improving the study of genetic contributors to AD. However, they do not account for the multifactorial nature of sporadic AD, which involves intricate gene–environment interactions [27].

**Table 14:** Strengths and weaknesses of different model systems used for AD research.

Model System	Strengths	Weaknesses	References
Transgenic Mouse Models	- Mimic key aspects of human AD (plaques, tangles, synaptic deficits) - Enable genetic manipulation and longitudinal studies	- Fail to replicate all human AD features (e.g., sporadic AD) - Differences in brain structure and ageing processes	[18]
3xTg-AD Mouse Model	- Simultaneously develops amyloid plaques and tau tangles - Provides insights into interactions between key pathologies	- Limited translation of therapies successful in mice to humans - Requires specific environmental and genetic factors to express pathology	[23]
Rat Models	- Better cognitive performance and more human-like brain complexity than mice - Suitable for pharmacokinetics and behavioural studies	- Lack of spontaneous AD pathology - Induced models only replicate specific aspects (e.g., amyloid injection)	[198]
*Drosophila* Models	- Short lifespan facilitates fast genetic studies - Inexpensive and very easy to handle	- Simplistic nervous system - Limited relevance to human AD pathology	[19]
*C. elegans* Models	- Simple, well-mapped nervous system - Suitable for high-throughput genetic screens and drug testing	- Limited complexity and lack of brain structure resembling humans	[24]
Non-Human Primates	- Share high genetic and physiological similarity with humans - Exhibit amyloid pathology similar to humans when aged	- High cost and ethical concerns - Slow disease progression and limited availability	[196]
iPSC-Derived Neurons	- Patient-specific models for studying sporadic and familial AD - High human relevance	- Lack of complexity of in vivo systems - Limited long-term culture viability	[25]
3D Brain Organoids	- Mimic structural and cellular complexity of the human brain - Suitable for studying amyloid and tau pathology	- Limited maturation and absence of vascular and immune systems	[26]
Blood–Brain Barrier on a Chip	- Provides insights into amyloid transport and drug delivery mechanisms	- Simplified system may not replicate full BBB complexity	[197]
CRISPR-Engineered Models	- Enable precise genetic modifications to study familial AD mutations	- Limited understanding of the interactions between genetic and environmental factors	[27]

## 6. Key Areas for Future Research

### 6.1. Developing Models for Late-Onset AD

Developing models for late-onset AD (LOAD) research requires a combination of genetic, pathophysiological, and computational approaches.

One widely used genetic model involves APOE4-knock-in mice, which incorporate the human APOE ε4 allele, a major genetic risk factor for LOAD. These mice exhibit retinal impairments, increased neuroinflammation, and the downregulation of synaptogenesis, making them valuable for studying retinal degeneration as a non-invasive biomarker for disease progression [199]. Another genetic approach includes Shugoshin 1 (Sgo1) haploinsufficient mice, which spontaneously develop Aβ accumulation, supporting the “three-hit hypothesis” that prolonged mitosis contributes to Aβ deposition and the development of LOAD [200].

Beyond genetic modifications, recent studies emphasise the role of autophagic and endolysosomal dysfunction in LOAD. The research suggests that genes involved in these pathways contribute to neurodegeneration, aligning well with the endolysosomal hypothesis of disease progression [201]. Additionally, computational modelling has provided new insights into biomarkers progression. Models simulating amyloid, tau, and neuronal loss have been developed to predict the LOAD evolution and potential responses to therapies, supporting the dynamic biomarker cascade theory [202].

Efforts to create robust animal models include initiatives like the MODEL-AD Consortium which integrates human genetic data to develop next-generation mouse models that better replicate the human LOAD pathology [195,203]. In addition, long-lived rodent models, such as *Octodon degus*, naturally develop a LOAD-like pathology, including cognitive decline, phospho-tau accumulation, Aβ deposition, and neuroinflammation. This species may offer a more accurate representation of LOAD compared to traditional short-lived, genetically modified models [204].

Advancements in imaging and ML are also transforming LOAD research. A radiomic analysis of the hippocampus has demonstrated high accuracy in distinguishing LOAD patients from healthy controls, highlighting its potential as a diagnostic biomarker [205]. Furthermore, ML techniques, such as a Support Vector Machine (SVM) and eXtreme Gradient Boosting (XGBoost), have shown promising results in predicting AD, reinforcing the role of AI in early detection and diagnosis [206].

Despite these advances, several challenges remain in modelling the complexity of LOAD. The disease is influenced by a combination of genetic and environmental factors, including epigenetic mechanisms and exposure to toxins, such as lead (Pb), which may play a role in disease onset and progression [207]. Future models must integrate various pathophysiological pathways, such as neuroinflammation, lipid metabolism, and amyloidogenic processes, to develop more comprehensive and translationally LOAD-relevant models [208].

In conclusion, the development of effective models for LOAD requires interdisciplinary collaboration, integrating genetic, computational, and biomarker-based approaches. The use of advanced animal models, ML, and initiatives like the MODEL-AD Consortium is essential for bridging the gap between preclinical research and clinical applications. Continued efforts to refine these models will be crucial for advancing our understanding and treatment of LOAD.

#### 6.1.1. Key Genetic Risk Factors for Late-Onset Alzheimer’s Disease

LOAD is influenced by multiple genetic factors. The most significant and well-established genetic risk factor is the APOE ε4 allele, which is strongly associated with an increased risk of developing LOAD and is considered the primary genetic marker for the disease [209,210,211,212,213,214,215]. However, LOAD is a complex disease, and additional genetic contributors have been identified through GWAS and whole-genome sequencing.

Among these, ABCA7 has been linked to cholesterol metabolism and cognitive decline, playing a significant role in neuronal health [216,217]. Similarly, BIN1, which is involved in endocytosis and synaptic function, has been implicated in AD progression [217,218]. Other genes, including CASS4, CD33, CD2AP, CELF1, CLU, CR1, DSG2, EPHA1, FERMT2, HLA-DRB5-DBR1, INPP5D, MS4A, MEF2C, NME8, PICALM, PTK2B, SLC24H4-RIN3, SORL1, and ZCWPW1, have been associated with key cellular processes, such as the immune response, lipid metabolism, and APP processing [209,214,216,217,218,219].

Beyond common genetic variants, rare mutations also play a role in LOAD susceptibility. For instance, TREM2, which is involved in the microglial function and immune response, has been identified as a risk factor for AD, particularly in relation to neuroinflammation [217,218]. Another rare variant, PLD3, has been detected through whole-genome sequencing and is believed to influence Aβ metabolism [217].

The genetic risk for LOAD is not solely dependent on individual genes, but rather on the interaction between multiple genetic factors. Studies suggest that interactions between APOE ε4 and other genes, such as ABCA7, may accelerate cognitive decline and neurodegeneration [216]. Furthermore, polygenic risk scores, which integrate the cumulative effect of multiple genetic variants, have proven to be more effective than single-gene approaches in predicting cognitive impairment and LOAD risk [220].

Beyond a genetic predisposition, environmental and vascular factors also contribute to LOAD susceptibility. Polymorphisms in genes related to vascular health, such as ACE1, may influence AD risk by affecting the cerebral blood flow and neurovascular function, demonstrating the complex interplay between genetic and environmental influences [221]. Additionally, genetic risk factors can vary based on the sex and APOE status, with evidence suggesting that certain gene–gene interactions have different effects in males and females [222].

In conclusion, while APOE ε4 remains the most prominent genetic risk factor for LOAD, recent advances in genetics have highlighted a broader network of genes that contribute to the disease. Table 15 summarises key genetic risk factors for LOAD. Understanding these genetic interactions and their relationship with environmental factors will be crucial in developing personalised strategies for LOAD prevention and treatment.

Understanding these genetic risk factors and their interactions are crucial for developing targeted therapies and improving diagnostic and prognostic tools for LOAD.

#### 6.1.2. Predicting the Onset of Late-Onset Alzheimer’s Disease Using Machine Learning Models

Machine learning (ML) models have shown significant potential in predicting the onset of late-onset Alzheimer’s disease (LOAD), using various data types and advanced algorithms. Neuroimaging data, such as MRI and PET scans, are commonly employed to train models that classify individuals at risk and predict disease progression over time [223,224,225,226]. Genetic information from GWAS and polygenic risk scores (PRS) enhances the prediction accuracy and helps identify novel genetic markers [227,228]. Clinical assessments, cognitive scores, and demographic data further improve ML-based risk stratification [229,230,231,232].

Several ML techniques have been applied effectively. Convolutional neural networks (CNNs) show a strong performance in analysing neuroimaging data [224]. Support vector machines (SVMs), often combined with regularisation methods like LASSO, are useful for feature selection and classification [226,228,233]. Ensemble methods, such as Random Forest and XGBoost, have achieved high accuracy by integrating multiple features [166,226], while regularisation approaches refine models by selecting the most relevant predictors [228,230].

These models report a high predictive performance, with neuroimaging-based models reaching 84.4% accuracy [224] and ensemble approaches achieving ROC values of up to 0.991 [226]. Genetic models have yielded AUC values around 0.84, which are further improved by combining PRS with other data types [228]. Metrics like RMSE and R^2^ confirm the effectiveness of boosting algorithms in predicting disease onset and progression [229].

Despite these advances, challenges remain. Integrating genetic, imaging, and clinical data is complex but vital to improving the model performance [232,234]. Ensuring consistent and trustworthy predictions is crucial for clinical adoption, with strategies like monotonicity constraints supporting stable risk estimates [225]. Importantly, the early identification of at-risk individuals could lead to more timely and effective interventions [229,235]. The continued development of ML models and comprehensive data integration will further enhance their clinical utility in diagnosing and managing AD.

### 6.2. Studying the Role of Neuroinflammation in AD Using Animal Models

To study the role of neuroinflammation in AD using animal models, researchers have employed various approaches to understand the complex interactions between neuroinflammatory processes and the AD pathology.

Neuroinflammation in AD is characterised by the activation of glial cells, particularly microglia and astrocytes, which are consistently observed in both rodent models and AD patients. These cells contribute to the neuroinflammatory environment by releasing pro-inflammatory cytokines and other mediators that exacerbate neuronal damage [236,237,238].

Transgenic mouse models have been developed to mimic chronic neuroinflammation. For example, models such as the GFAP-IL6 mouse, which overexpress interleukin-6 (IL-6) in astrocytes, exhibit progressive neurodegeneration and cognitive decline, making them valuable models for studying the effects of neuroinflammation on AD progression [239].

Moreover, the activation of inflammasomes, such as the NLRP3 inflammasome, has been shown to play a significant role in neuroinflammation and neuronal death in AD models. Aβ can accelerate neuroinflammatory cell death through inflammasome activation, highlighting potential therapeutic targets [240,241]. Anti-inflammatory treatments, like minocycline have been tested in AD-like mouse models and demonstrated a reduction in microglial activation alongside improvements in memory, suggesting the potential of such therapeutic agents [240].

Additionally, novel animal models, such as the acrolein-induced sporadic AD mouse model have been developed to better replicate key pathological features of AD, including neuroinflammation, synaptic damage, and cognitive impairments. These models are crucial for elucidating disease mechanisms and testing new treatments [242].

However, challenges remain in translating findings from animal models to human AD due to species-specific differences in the brain complexity and disease manifestation [236,238,239]. Advances in imaging techniques, such as positron emission tomography (PET) and magnetic resonance spectroscopy (MRS), are increasingly applied to non-invasively track neuroinflammation in vivo, thereby supporting early AD diagnosis and the monitoring of therapeutic responses [243].

In short, animal models are indispensable tools for studying the role of neuroinflammation in AD. They help to elucidate underlying mechanisms and to provide platforms for testing potential therapeutic interventions. Nevertheless, the complexity of AD necessitates the continuous development and refinement of these models to enhance their relevance and translatability to human AD.

#### Key Inflammatory Markers in AD Animal Models

Elevated levels of interleukins (IL-1β, IL-6, and IL-10) have been observed in the cerebral tissue of AD mice, indicating their role in disease progression [244,245]. In particular, IL-6 has been noted for its dual role in promoting neuron survival as well as inducing neurodegeneration and apoptosis [245]. Tumour necrosis factor-α (TNF-α) is a potent pro-inflammatory cytokine commonly elevated in AD models; it exacerbates neuroinflammation and has emerged as a proper target for therapeutic interventions [244,245,246].

Monocyte chemoattractant protein-1 (MCP-1) is associated with tau pathology and correlates well with neuroinflammatory responses in AD models [247,248,249]. Similarly, YKL-40, a marker linked to glial activation, has shown strong associations with AD pathology, including elevated tau levels and cognitive decline [250,251]. Furthermore, the triggering receptor expressed on myeloid cell 2 (TREM2), which is involved in microglial activation, has been found to be reduced in aged AD rat models and to correlate with cognitive decline [252].

Markers of vascular injury and angiogenesis, such as vascular endothelial growth factor (VEGF) and its receptor VEGFR-1, are also implicated in AD and have been linked to the tau pathology in animal models [248]. In the context of glial cell activation, the GFAP-IL6 mouse model, which overexpresses IL-6 in astrocytes, demonstrates chronic neuroinflammation accompanied by significant neurodegeneration and cognitive decline, highlighting the pivotal role of astrocyte-derived IL-6 in AD [239]. Additionally, toll-like receptor 4 (TLR4) is involved in mediating the microglial response to amyloid plaques and stimulating the production of pro-inflammatory cytokines, such as TNF-α and IL-6 [253]. Table 16 summarises the key role of inflammatory mediators in AD research.

These markers collectively highlight the complex interplay among cytokines, chemokines, and other inflammatory mediators in the neuroinflammatory processes of AD, providing potential targets for therapeutic interventions.

### 6.3. Addressing the Genetic and Environmental Complexity of Human AD

To address the genetic and environmental complexity of human AD research, it is essential to consider both genetic predispositions and environmental influences that contribute to AD pathogenesis.

#### 6.3.1. Genetic Factors

Genetic Variants: AD is influenced by numerous genetic factors. Key genes associated with AD include *APOE*, *APP*, *PSEN1*, and *PSEN2*, which are linked to both early-onset and late-onset forms of the disease [254,255]. GWAS have identified several single-nucleotide polymorphisms (SNPs) in genes, such as *ABCA7*, *BIN1*, and *CD33* that are associated with increased AD risk [254,256].

#### 6.3.2. Epigenetic Mechanisms

Epigenetic changes such as DNA methylation play a significant role in AD. These changes can be influenced by environmental factors and age, affecting gene expression without altering the DNA sequence [257,258]. Studies have shown differential methylation patterns in AD patients, which may contribute to disease progression [258].

#### 6.3.3. Environmental Factors

##### Modifiable Risk Factors

Environmental factors, such as one’s diet, lifestyle, exposure to pollutants, and education level, have been implicated in AD risk. For instance, exposure to heavy metals, like aluminium and lead, as well as pesticides can increase the risk of AD by promoting Aβ accumulation and tau hyperphosphorylation [259,260,261].

##### Lifestyle and Diet

Physical activity, cognitive engagement, and healthy diet patterns are associated with a reduced risk of AD. These factors can modulate genetic predispositions and potentially delay the onset of AD [260,262].

##### Microbiome Influence

Emerging research suggests that the gut microbiome and viral infections may contribute to AD through mechanisms involving neuroinflammation and immune responses [263].

#### 6.3.4. Gene–Environment Interactions

##### Complex Interactions

The interplay between genetic predispositions and environmental exposures is complex. For example, carriers of certain genetic variants may experience different levels of cognitive decline based on their environmental exposures, such as diet and pollutants [264,265,266].

##### Epigenetic Modifications

Environmental factors can induce epigenetic modifications that affect the expression of AD-related genes. These modifications can occur at critical periods, such as early life, and have long-lasting effects on disease risk [266,267].

#### 6.3.5. Research Implications

##### Comprehensive Approaches

To fully understand AD, it is crucial to adopt comprehensive research approaches that consider both genetic and environmental factors. This includes studying diverse populations and employing advanced technologies, like GWAS and epigenome-wide association studies (EWAS) [264,265,268].

##### Preventive Strategies

Identifying modifiable environmental risk factors provides opportunities for preventive interventions. Lifestyle modifications, such as increased physical activity and dietary changes, could potentially mitigate genetic risks and delay the onset of AD [260,262].

In summary, AD research must integrate genetic, epigenetic, and environmental perspectives to unravel the complex aetiology of the disease. This holistic approach can lead to more effective AD prevention and treatment strategies.

### 6.4. Ethical Considerations in Developing and Using Animal Models in AD Research

Developing and using animal models for AD research involves several ethical considerations that must be carefully addressed to ensure responsible and humane scientific practices.

#### 6.4.1. Species Selection and Ethical Constraints

While primates, dogs, and bears can naturally develop an AD-like pathology, their use is largely limited due to ethical, availability, and economical reasons [269,270]. Commonly used laboratory animals like mice and rats do not naturally develop AD, but can be genetically modified to mimic certain aspects of the disease [269,270]. The development of transgenic models, such as mice expressing human AD-related genes, has been a significant scientific advance. However, these models often only partially replicate the human AD condition, raising questions about their validity and ethical justification [9,14].

#### 6.4.2. Validity and Predictive Power

The ethical use of animal models requires that they have face, construct, and predictive validity. This means the model should closely mimic human disease pathology, involve similar biological mechanisms, and respond to treatments in ways that predict human AD outcomes [13,271]. No current animal model fully replicates AD, which challenges their ethical justification. The lack of success in translating findings from animal models to human treatments underscores the need for better animal models and a careful consideration of their limitations [13,14,196].

#### 6.4.3. Minimising Harm and Suffering

Ethical research mandates the refinement of experimental protocols to minimise animal suffering. This includes improving housing conditions, using less invasive techniques, and ensuring proper pain management [269,272]. Researchers are encouraged to reduce the number of animals used and to replace animal models with alternative methods, whenever possible. This aligns totally with the principles of the 3Rs (Replacement, Reduction, and Refinement) in animal research ethics [269,272].

#### 6.4.4. Gender Considerations

Both male and female animals should be used to understand gender-specific differences in the AD pathology and treatment responses. This is crucial for developing comprehensive AD therapeutic strategies [270,272].

#### 6.4.5. Responsibility and Oversight

Researchers bear the responsibility for the ethical use of animals in AD research. This includes justifying the choice of animal models, ensuring humane treatment, and adhering strictly to ethical guidelines and regulations [269].

## 7. Clinical Implications of Research Models

### 7.1. Translating Preclinical Findings to Human Clinical Trials in AD Research

Translating preclinical findings to human clinical trials in AD research presents numerous challenges. Despite significant advances in understanding AD, the failure rate of clinical trials remains high, largely due to limitations in animal models, methodological issues, and the complexity of AD pathogenesis. Addressing these challenges requires a multi-faceted approach that includes improving preclinical models, enhancing study designs, fostering collaboration, and ensuring ethical considerations are met.

One of the primary challenges in AD research is the limited translatability of findings from animal models. While these models have been invaluable for studying disease mechanisms and testing treatments, they fail to fully replicate human AD pathology, contributing to the frequent discrepancies between preclinical successes and clinical trial failures [54,273,274]. Variability in breeding practices, colony maintenance, and the absence of standardised protocols further reduce the reliability and reproducibility of preclinical results [273,274]. Methodological shortcomings, including inadequate scientific rigor and reproducibility, contribute significantly to high failure rates in clinical trials [275,276]. Publication bias and the under-reporting of negative or inconclusive findings further distort perceptions of treatment efficacy, misguiding research priorities and resource allocation [273].

The complexity of AD pathogenesis compounds these issues. For years, the field has been dominated by the amyloid hypothesis, often overshadowing alternative disease mechanisms that may be critical to effective treatment [277]. Most current models also fail to accurately represent early-stage pathologies seen in humans, limiting their usefulness in identifying optimal therapeutic windows [274].

To address these limitations, the development of improved preclinical models and more rigorous experimental strategies is essential. This includes using go/no-go decision points to reduce bias, confirming pharmaceutical ingredient integrity, and adhering to established guidelines like ARRIVE to enhance the study quality [275,276]. Comprehensive study designs with clearly defined hypotheses, prior pharmacokinetics–pharmacodynamics (PK/PD) analysis, and the incorporation of biomarkers can significantly strengthen the predictive value of preclinical studies [273]. Ensuring robust sample sizes is also key to improving statistical power and reliability.

Collaboration within the research community plays a crucial role in improving translation. Initiatives like the MODEL-AD consortium and dedicated translational workshops provide critical training, standardised tools, and shared resources to facilitate progress [276,278]. Establishing public repositories for all study outcomes—positive, negative, or neutral—can further mitigate the publication bias and enhance transparency.

Ethical and practical considerations must also be addressed. Engaging patients through effective recruitment and retention strategies is vital for clinical trial success [277,279,280]. Furthermore, ethical concerns related to early diagnosis and interventions should be considered to align research with patient-centred goals.

Overall, improving translation from preclinical models to clinical outcomes in AD research depends on refining experimental approaches, enhancing collaboration, ensuring transparency, and upholding ethical standards. These combined efforts will help advance the development of reliable and effective AD therapies.

### 7.2. Optimising Therapeutic Strategies Based on Research Models’ Findings

To optimise therapeutic strategies for Alzheimer’s disease (AD), it is essential to acknowledge its multifactorial nature and apply insights from diverse research models. Targeting early-stage metabolic dysfunction—such as mitochondrial deficits and brain hypometabolism—can help prevent decline and promote healthy ageing. Enhancing glucose metabolism and supporting mitochondrial function, including through ketogenic interventions during the prodromal phase, has shown promise in delaying cognitive decline [281].

The early identification of at-risk individuals is critical to prevent irreversible neuronal damage. Predictive modelling plays a key role in this effort, enabling the application of preventative strategies during early disease stages [282,283]. Moreover, personalised, multi-therapeutic approaches that address factors like age, sex, diet, stress, and nutrient deficiencies outperform single-drug therapies by supporting broader network functions and improving cognitive outcomes [284,285,286].

Drug repurposing offers an efficient path to treatment development. Agents like bumetanide and fingolimod have shown the ability to reverse cognitive deficits in AD models [287,288], while others, such as dextromethorphan, pimavanserin, and cannabinoids, target neuropsychiatric symptoms, offering symptomatic relief and potential disease modification [289].

Finally, ethical concerns surrounding early intervention and predictive modelling must be addressed to ensure equitable access and patient protection [283]. Innovative clinical trial designs, coupled with cross-sector collaboration among academia, industry, and public agencies, are vital for advancing therapeutic development [290]. Collectively, these strategies support the move toward more effective, personalised interventions that can improve outcomes and quality of life for AD patients.

## 8. Conclusions and Recommendations

### 8.1. Summary of Key Insights

This document reviews the use of mouse and alternative models in AD research, emphasising their contributions, limitations, and future directions. Animal models are essential for studying AD pathogenesis and testing novel treatments, with transgenic mouse models widely used for replicating Aβ plaques and tau tangles. However, these models do not fully capture the complexity of human AD, particularly late-onset forms. Knock-in models avoid overexpression artefacts, improving physiological relevance, while injection models induce an AD-like pathology, but are invasive.

To address these limitations, alternative models have been developed. Zebrafish provide genetic similarities to humans and are useful for drug screening. *D. melanogaster* and *C. elegans* allow genetic manipulation for studying amyloid toxicity and molecular pathways. Marmosets, as non-human primates, share closer genetic and pathological traits with humans, enhancing the translational potential. In vitro models, such as human brain organoids, offer controlled environments for studying AD mechanisms, but lack systemic interactions.

Advances in imaging techniques, ML, and omics technologies have improved biomarker detection and analysis. While mouse models are effective for early-stage research, alternative models, especially non-human primates and organoids, offer better human AD representation. However, scalability and ethical concerns remain major challenges. Future research should focus on LOAD models, neuroinflammation, genetic–environmental interactions, and ethical considerations.

Clinically, bridging the gap between preclinical models and human trials is crucial for continued and successful AD research. Optimising therapeutic strategies and developing non-invasive biomarkers will enhance early AD diagnosis and treatment. Integrating multiple models and refining the existing ones will improve the research accuracy and accelerate the development of effective AD novel therapies.

### 8.2. Recommendations for Future Research Directions

Future research on AD should focus on developing models that better represent LOAD, which accounts for most of the human cases. *APOE4*-knock-in mice are valuable for studying genetic risk factors, as they exhibit increased neuroinflammation and synaptic dysfunction. Additionally, alternative species, like *Octodon degus*, which naturally develop an AD-like pathology, could provide more accurate representations of LOAD. Expanding the genetic diversity in mouse models is also vital for capturing molecular heterogeneity and improving the translational relevance. Integrating behavioural assessments and biomarkers will enhance the reliability, while multi-model approaches incorporating non-human primates and alternative species could offer deeper insights.

Neuroinflammation, a key driver of AD progression, should be further investigated using models such as GFAP-IL6 transgenic mice to study chronic inflammation’s impact on neurodegeneration. Targeting inflammasome activation, particularly NLRP3, may provide new therapeutic opportunities. Alternative models, such as zebrafish, enable the high-throughput screening and real-time visualisation of disease progression. *D. melanogaster* and *C. elegans* models allow for rapid genetic manipulation to study the amyloid toxicity. Non-human primates like marmosets offer greater physiological similarity to humans, while 3D brain organoids provide structural complexity, though they lack vascular and immune systems.

Advancing computational and imaging technologies will improve AD research. ML and AI can enhance early diagnosis and biomarker discovery, while advanced imaging techniques, such as phase-contrast X-ray CT (PCXCT) and PET scans, offer the non-invasive detection of amyloid plaques. Ethical considerations must also be prioritised in clinical trials design, with greater focus on AD patient engagement and equitable access to emerging treatments. Optimising therapeutic strategies should include targeting mitochondrial bioenergetic deficits and integrating multi-therapeutic approaches, such as sex, age, dietary, lifestyle, and pharmaceutical interventions. Repurposing FDA-approved drugs, like bumetanide and fingolimod may accelerate treatment development.

Future AD research should embrace a multi-model approach that integrates genetic, environmental, and neuroinflammatory factors. Advances in technology, ethics, and therapeutics will be essential for translating with success preclinical discoveries into effective human AD treatments.

### 8.3. Potential Impact on AD Treatment and Prevention

AD is a growing global health concern, affecting millions worldwide. Research advances outlined in this document highlight key strategies that could significantly impact AD future treatment and prevention efforts.

Repurposing FDA-approved drugs has shown promising results in AD treatment. Medications such as bumetanide and fingolimod target specific pathways implicated in AD pathology and have demonstrated therapeutic potential. Additionally, novel pharmacological agents, like dextromethorphan formulations, pimavanserin, and cannabinoids, are being exploited for their ability to manage neuropsychiatric symptoms and modify disease progression.

Early intervention is critical in AD management, and predictive modelling plays a key role in identifying individuals at-risk. ML techniques are improving AD risk prediction by integrating genetic, neuroimaging, and clinical data. Advanced imaging technologies, such as phase-contrast X-ray CT (PCXCT) and PET scans, enhance biomarker detection, further supporting early AD diagnosis and intervention strategies.

Given the complexity of AD, a single-drug approach may not be sufficient. A multi-therapeutic strategy combining lifestyle modifications, dietary interventions, and pharmacological treatments is being exploited. Targeting mitochondrial bioenergetic deficits and brain hypometabolism is crucial for preventing early metabolic decline, while supplementing with ketone bodies may help sustain cognitive function in early-stage AD.

As predictive models and early diagnostic tools improve, ethical concerns regarding access to treatment and preventive measures must be addressed. Equitable recruitment strategies and the fair distribution of emerging therapies are necessary to ensure that all AD patients benefit from the most recent advances. Bridging the gap between preclinical research and human clinical trials remains a major challenge, requiring collaboration among academic institutions, government agencies, and pharmaceutical companies besides society to accelerate drug development. Developing robust animal models that better replicate human AD pathology can improve the success rate of clinical trials.

Future advancements in AD treatment will rely on a combination of drug repurposing, predictive modelling, multi-target therapies, and ethical considerations. Investing in early AD diagnosis, personalised interventions, and translational research will be essential to improve patient outcomes and slowing or halting AD progression.

## Figures and Tables

**Table 1:** Timeline of models in AD research.

Year	Milestone	Main Findings	References
1980s	Cholinergic Deficit Models	Early models using AF64A and ibotenic acid targeted cholinergic neurons to mimic cognitive deficits observed in AD	[20]
1995	PDAPP Mouse Model (transgenic)	The first transgenic mouse model with human APP mutations linked to familial AD showed amyloid plaques and synaptic deficits	[21]
1998	*Drosophila* APP Models	Demonstrated γ-secretase cleavage and amyloid pathology in *D. melanogaster* expressing human APP	[22]
1999	“Alzheimer’s Vaccine” in Mice	Immunisation with β-amyloid prevented amyloid plaque development in APP-transgenic mice	[23]
2000	APP/PS1 Double-Transgenic Model	Developed mice expressing human APP and PSEN1 mutations; these showed accelerated amyloid deposition and cognitive decline	[24]
2001	Tau Transgenic Mice (rTg4510)	Introduced mice expressing human tau mutations, leading to tangles, neuronal loss, and behavioural impairments	[25]
2005	3xTg-AD Mouse Model	Combined APP, PSEN1, and tau mutations to create a mouse model with both plaques and tangles, mimicking human AD pathology	[26]
2010	*C. elegans* Models	Expressed human β-amyloid or tau, exhibiting aggregation and neuronal dysfunction, useful for genetic studies of AD	[27]
2012	iPSC-Derived Neuronal Models	First use of induced pluripotent stem cells (iPSCs) from AD patients to create neuronal cultures showing AD-like pathology in vitro	[28]
2014	Organoids for AD Research	Developed 3D brain organoids from human stem cells to study amyloid and tau pathology	[29]
2015	Non-Human Primate Models	Created aged rhesus monkeys and marmosets with amyloid-β infusions, showing plaques and synaptic dysfunction similar to human AD	[11]
2019	CRISPR-Engineered Mouse Models	Applied CRISPR-Cas9 to introduce precise mutations in APP and tau genes, improving modelling accuracy for familial AD	[30]
2020	Blood–Brain Barrier (BBB) on a Chip	Developed microfluidic BBB models for studying amyloid transport and drug delivery in vitro	[31]
2022	Humanised Mouse Models	Generated transgenic mice carrying humanised APP and tau genes, providing more accurate disease progression and therapeutic response data.	[32]

**Table 2:** Most common transgenic murine models for AD research.

Murine Model	Genetic Modifications	Key Features	References
5xFAD	APP with three FAD mutations	Amyloid plaques and subsequent neurodegeneration	[44,45]
APPswe/PS1dE9	APPswe + PSEN1dE9	Amyloid plaques, cognitive deficits	[46]
Tg2576	APPswe	Amyloid plaques, behavioural changes	[47]
3xTg-AD	APPswe + PSEN1M146V + tauP301L	Amyloid plaques, tau tangles, cognitive impairments	[48]
APP/PS1	Various APP and PS1 mutations	Enhanced amyloid pathology	[49]

**Table 3:** Comparison of the specified mutations involved in FAD commonly used in murine models.

Mutation	Gene	Key Features	Animal Models	Pathology	References
London	APP	V717I mutation, increases Aβ production	Mouse	Amyloid plaques, cognitive impairment	[39]
Florida	APP	I716V mutation, increases Aβ production	Mouse	Amyloid plaques, cognitive impairment	[39]
Iberian	APP	I716F mutation, increases Aβ production	Mouse	Amyloid plaques, cognitive impairment	[39]
Austria	APP	T714I mutation, increases Aβ production	Mouse	Aβ accumulation and brain atrophy	[63]
Flemish	APP	A692G mutation, increases Aβ production	Mouse	Behavioural disturbances without amyloid deposits, glial activation, and microspongiosis	[64]
Swedish	APP	K670N/M671L mutation, increases Aβ production	Mouse (e.g., Tg2576)	Early amyloid plaques, cognitive impairment	[38,39]
Iowa	APP	D23N mutation, increases Aβ aggregation	Mouse	Accelerated Aβ aggregation	[65]
Arctic	APP	E22G mutation, increases Aβ aggregation	Mouse	Resistant to proteolytic degradation, prone to aggregation	[39,65]
Dutch	APP	E22Q mutation, increases Aβ aggregation	Mouse	Accelerated Aβ aggregation	[65]
Indiana	PSEN1	P117L mutation, increases Aβ production	Mouse	Early amyloid deposition, neuroinflammation	[39]
Indiana	PSEN1	ΔE9 mutation, increases Aβ production	Rat (e.g., TgF344-AD)	Accumulation of Aβ plaques increasing with age	[66]

APP Mutations: These mutations have been shown to increase the production or aggregation of Aβ, which is known to lead to the formation of amyloid plaques. These plaques are a hallmark of AD pathology. Examples include the Swedish, Arctic, and Dutch mutations [39,65]. PSEN1 Mutations: nutations in the presenilin 1 gene, such as the Indiana mutation, are also used in models to study early amyloid deposition and neuroinflammation [39].

**Table 4:** Comparison of knock-in and injection models.

Feature	Knock-In Models	Injection Models
Physiological Relevance	High, as they avoid artefacts’ overexpression [67,68,69]	Moderate, as they can target specific brain regions but are invasive [74,75,76]
Pathological Characteristics	Amyloid plaques, neuroinflammation, synaptic dysfunction [29,67,73]	Induced amyloid pathology, neurotoxicity [74,75,76]
Behavioural Deficits	Age-dependent cognitive impairments [67,71,72,73]	Acute and chronic memory impairments [74,75]
Tau Pathology	Generally absent [69]	Not typically induced [74,75]
Invasiveness	Low [67,68,69]	High [74,75,76]
Use in Long-Term Studies	Suitable for long-term studies [67,71,72,73]	More suitable for short-term studies [74,75]

**Table 5:** Key findings of murine models for understanding AD athogenesis.

Model	Key Findings	References
APPswe/PS1dE9	Revealed neuronal hyperexcitability and synaptic dysfunction, consistent with human AD studies	[77]
3xTg-AD	Demonstrated interaction between Aβ and tau, driving AD pathogenesis	[78,84]
J20	Showed early memory impairment and neuroinflammatory responses before Aβ deposition	[82]
5xFAD	Enhanced endocannabinoid tone improved memory and reduced neuroinflammation	[83]
APP23	Provided insights into cognitive impairments and behavioural changes	[10,11]
FAD4T	Highlighted the role of microglia and synaptic pruning in AD pathogenesis	[85]

**Table 6:** Limitations and challenges of murine models in AD research.

Limitation/Challenge	Description	References
Incomplete Recapitulation	Models do not fully mimic human AD pathology	[54,90,91,92]
Species-Specific Differences	Differences in brain anatomy and physiology	[54,91,94]
Oversimplification	Focus on amyloid hypothesis, neglecting other factors like tau pathology and neuroinflammation	[91,96]
Translational Failures	Preclinical success does not translate to clinical trials	[54,95,97]
Behavioural Discrepancies	Inconsistent cognitive and behavioural assessments	[96,98,99]
Model Selection	Difficulty in choosing and validating appropriate models	[96,98]
Technological Advances	Need for integrating new technologies and methods	[95,100]
Ethical Considerations	Ethical and practical limitations in research	[101]

**Table 7:** Characteristics of marmoset models for AD research.

Feature	Details	References
Genetic Similarity	Closer to humans than rodents, enabling study of primate-specific mechanisms	[134,135]
Natural AD Pathologies	Develop Aβ plaques and tau abnormalities naturally	[135,136,137]
Lifespan	Short lifespan allows for feasible longitudinal studies	[136,138]
Biomarkers	Key biomarkers identified for neural degeneration	[139]
Tau Protein	Express 3R and 4R tau isoforms, similar to humans	[137]
Neuroinflammation	Immune system’s role in amyloid plaque formation studied	[140]

**Table 8:** In vitro models for AD research.

Model Type	Advantages	Limitations	References
2D Cell Cultures	- Simple and scalable - Useful for high-throughput screening	- Lack complexity - Poor mimicry of in vivo environment	[142,143]
3D Cell Cultures/Organoids	- Enhanced physiological relevance - Mimic brain structure and function	- Complex and costly - Incomplete mimicry of the human brain	[141,146,147,149,152]
Co-culture Systems	- Improved cell–cell interaction studies - Simpler than full organoids	- Still more complex than 2D cultures - May not fully replicate in vivo conditions	[151]
Microfluidic Devices	- Dynamic and versatile - Real-time analysis	- Technical complexity - High cost	[153,154]

**Table 9:** Advantages and disadvantages of alternative models for AD research.

Model	Advantages	Disadvantages
Zebrafish	- High genetic similarity to humans (90%) [155]	- Requires specialised monitoring systems [156]
	- Low cost and high fertility rate [156]	- Limited use in current drug discovery pipelines [157]
	- Amenable to high-throughput screening [157,158]	- Translational gap still exists despite advantages [155]
	- External development allows easy observation [155]	
	- Suitable for genetic manipulation and transgenesis [159]	
	- Can model neurodegenerative diseases like AD [155]	
*Drosophila*	- Low cost and easy to maintain [158]	- Less genetic similarity to humans compared to vertebrates [158]
	- Amenable to high-throughput genetic studies [158]	- Limited complexity in modelling human diseases [158]
	- Short life cycle and rapid generation time [158]	
*C. elegans*	- Simple and well-understood nervous system [158]	- Even less genetic similarity to humans than Drosophila [158]
	- Transparent body allows easy observation [160] (Youssef et al., 2018)	- Limited behavioural complexity [158]
	- Low cost and easy to maintain [158]	
Marmoset	- Closer genetic and physiological similarity to humans [156]	- High cost and ethical concerns [156]
	- Can model complex behaviours and cognitive functions [156]	- Longer life cycle and lower reproductive rate [156]
In Vitro	- Allows detailed molecular and cellular studies [161]	- Lack of whole-organism context [161]
	- Can use human cells directly, reducing translational gap [161]	- Limited ability to model complex interactions and behaviours [161]
	- Amenable to high-throughput screening [161]	

**Table 10:** Key features and applications of advanced imaging techniques in AD research.

Technique	Key Features	Applications in AD Research	Advantages	Limitations	References
X-ray Phase Contrast Tomography (XPCT)	- Non-destructive 3D imaging	- Visualises amyloid plaques and vascular networks in AD mouse brains	- Preserves tissue chemistry and structure	- Currently limited to ex vivo studies	[162]
	- High spatial and contrast resolution	- Detailed visualisation of amyloid angiopathy at capillary level	- Enables comparison of physiological vs. pathological states	- Requires high radiation doses	
Phase-Contrast X-Ray-Computed Tomography (PCXCT)	- High sensitivity	- Detects high-density amyloid plaques	- High sensitivity comparable to histological analysis	- Limited to postmortem samples	[163]
	- Non-invasive	- Quantifies amyloid plaque load	- Provides 3D visualisation	- High radiation exposure	
	- No imaging agents required	- Reveals age-related changes in plaque density			
Diffraction-Enhanced Imaging (DEI)	- Greater soft tissue contrast	- Visualises small amyloid plaques in AD mouse brains	- High resolution	- Proof of principle stage	[164]
	- Higher resolution than MRI	- Identifies anatomical structures like hippocampal subregions	- Effective for early diagnosis	- Requires further validation	
Analyser-Based X-ray Imaging (ABI)	- High contrast and spatial resolution	- Potential for in vivo applications	- High sensitivity	- Technical challenges in in vivo imaging	[165]
	- Uses monochromatic X-rays	- Detailed depiction of anatomical structures	- Low radiation dose	- Limited current applications	

**Table 11:** Machine learning approaches for AD prediction.

Approach	Techniques	Data Types	Performance	Key Insights	References
Supervised Learning	CatBoost, Logistic Regression, Decision Tree, Random Forest, Naїve Bayes, SVM, Gradient Boosting, XGBoost, AdaBoost	Clinical, MRI	Accuracy: 92–96%	CatBoost, SVM, and Decision Tree performed best	[166]
Neuroimaging Analysis	SVM, Decision Tree, Logistic Regression, Random Forest, CNN, VGG16	MRI, PET	Accuracy: SVM 79%, Random Forest 67%, CNN 87%, VGG16 91%	VGG16 outperformed traditional ML algorithms	[167]
Genetic Data Analysis	Various ML algorithms	Genetic Data	AUC: 0.59–0.98	High risk of bias due to feature selection and validation methods	[168]
Multi-Modal Analysis	Multi-Task Model, Time Series model, Deep Learning	Clinical, Genetic, Neuroimaging	Not specified	Emphasises combining diverse biomarkers for better prediction	[169,170]
Ensemble Methods	Random Forest, XGBoost, Gradient Boosting, CatBoost, Voting Classifier	Clinical, Cognitive Tests	Accuracy: 96.30%	Ensemble methods highly effective	[171]
Hybrid Models	Stacking (Logistic Regression, Naїve Bayes, SVM, Decision Trees, Random Forest, XGBoost)	Cognitive, Demographic	Accuracy: 95.12%	Hybrid models improved prediction accuracy	[172]
Deep Learning	CNN, Transfer Learning (VGG16)	Neuroimaging	Accuracy: CNN 87%, VGG16 91%	Transfer learning models show high potential	[167]
Non-Invasive Techniques	KNN, Naїve Bayes, ANN	Cognitive Tests	F1-Scores: KNN 0.7, Naїve Bayes 0.88, ANN 0.96	ANN showed highest F1-Score	[173]

**Table 12:** Omics technology in AD research.

Omics Technology	Application	Insights	Biomarkers	References
Metabolomics	Diagnosis, Drug Evaluation	Mimics human metabolic changes	LysoPC, sphingolipid intermediates	[176]
Proteomics	Biomarker Discovery	Reflects protein alterations in CSF and plasma	Various protein markers	[177,178]
Transcriptomics	Gene Expression Analysis	Identifies mRNA and miRNA changes	mRNA, miRNA predictors	[177,183]
Genomics	Genetic Risk Factors	SNP analysis for genetic insights	SNPs, gene expression data	[183]

**Table 13:** Comparative on translational discrepancies between AD models.

Feature	Mouse Models	Alternative Models
Common Models	Transgenic models (e.g., 5XFAD, APP23, Tg2576, 3xTg) [184,185,190,191].	Degu, Dog, Non-human Primates, *C. elegans*, *D. melanogaster*, *D. rerio* [187,188].
Pathological Characteristics	Amyloid plaques, neurofibrillary tangles, cognitive deficits, and neuroinflammation [67,134,184,185,190].	Varies among species; some recapitulate neuropathology and cognitive impairments better [187,188].
Translational Success	Limited; many preclinical successes do not translate to clinical human efficacy [54,184,192].	Potentially higher translational value due to closer resemblance to human pathology in some models [187,188].
Genetic Manipulation	Extensive use of genetic modifications to induce AD-like symptoms [67,134,184,185].	Less genetic manipulation; some models naturally develop AD-like symptoms [187,193].
Behavioural Assessments	Cognitive tests (e.g., Morris water maze, Y-maze, Barnes maze) [67,190,194].	Varies; some models may not be suitable for traditional rodent cognitive tests [187,188].
Molecular Similarities	Transcriptomic and proteomic similarities with human AD subtypes [189,195].	Some models share core molecular programs with mouse models [188].
Limitations	Do not fully replicate human AD; variability in pathology and behaviour [54,184,192].	Some models may not develop all AD features; variability in results [193].
Innovative Approaches	Knock-in models, a combination of multiple transgenic lines [67,134].	Use of naturally occurring models, cross-species comparisons [187,188].

**Table 15:** Key genetic risk factors for late-onset AD.

Gene	Role/Function	Association with LOAD	References
*APOE ε4*	Lipid transport, amyloid processing	Strongest genetic risk factor	[209,211]
*ABCA7*	Cholesterol metabolism	Cognitive decline, LOAD risk	[216,218]
*BIN1*	Endocytosis, synaptic function	LOAD risk	[212,217]
*TREM2*	Microglial function, immune response	Increased risk, rare variant	[214,215]
*PLD3*	Unknown	Increased risk, rare variant	[213]
*PICALM*, *CR1*	Various cellular processes	LOAD risk	[219,221]
*ACE1*	Vascular health	Potential risk factor	[222]

**Table 16:** Role of inflammatory markers in AD research.

Marker	Role in AD	References
IL-1β, IL-6, IL-10	Pro-inflammatory cytokines, neurodegeneration	[244,245]
TNF-α	Potent pro-inflammatory cytokine, exacerbates neuroinflammation	[244,245,246]
MCP-1	Associated with tau pathology, neuroinflammation	[247,248,249]
YKL-40	Glial activation, associated with tau levels and cognitive decline	[250,251]
TREM2	Microglial activation, reduced in aged, impaired AD rats	[252]
VEGF, VEGFR-1	Vascular injury, associated with tau pathology	[248]
GFAP-IL6	Chronic neuroinflammation, neurodegeneration	[239]
TLR4	Microglial response to amyloid plaques, cytokine production	[253]

## Abbreviations

The following abbreviations are used in this manuscript:

ADNI	Alzheimer’s Disease Neuroimaging Initiative
ABI	Analyser-Based X-Ray Imaging
AD	Alzheimer’s Disease
ADNI	Alzheimer’s Disease Neuroimaging Initiative
AI	Artificial Intelligence
APOE	Apolipoprotein E
APP	Amyloid Precursor Protein
AUC	Area Under the Curve
Aβ	Beta-Amyloid
BBB	Blood–Brain Barrier
CNNs	Convolutional Neural Networks
CRISPR	Clustered Regularly Interspaced Short Palindromic Repeats
CSF	Cerebrospinal Fluid
*C. elegans*	*Caenorhabditis elegans*
DEI	Diffraction-Enhanced Imaging
*D. melanogaster*	*Drosophila melanogaster*
EWAS	Epigenome-Wide Association Studies
FAD	Familial Alzheimer’s Disease
FDA	Food and Drug Administration
GFAP	Glial Fibrillary Acidic Protein
GWAS	Genome-Wide Association Studies
hAPP	Human Amyloid Precursor Protein
ICV	Intracerebroventricular
IL-6	Interleukin-6
IL-10	Interleukin-10
iPSCs	Induced Pluripotent Stem Cells
LC-MS	Liquid Chromatography–Mass Spectrometry
LOAD	Late-Onset Alzheimer’s Disease
LysoPC	Lysophosphatidylcholine
MAPT	Microtubule-Associated Protein Tau
MCP-1	Monocyte Chemoattractant Protein-1
ML	Machine Learning
MMSE	Mini-Mental State Examination
MRS	Magnetic Resonance Spectroscopy
NfL	Neurofilament Light Chain
NLRP3	NOD-Like Receptor Protein 3
PCXCT	Phase-Contrast X-Ray-Computed Tomography
PET	Positron Emission Tomography
PK/PD	Pharmacokinetics–Pharmacodynamics
PRS	Polygenic Risk Scores
PSEN	Presenilin
qRT-PCR	Real-Time Quantitative Reverse Transcription PCR
RMSE	Root Mean Squared Error
R^2^	R-Squared
SCD	Stearoyl CoA Desaturase
SNP	Single-Nucleotide Polymorphism
SVM	Support Vector Machine
TLR4	Toll-Like Receptor 4
TNF-α	Tumour Necrosis Factor-Alpha
TREM2	Triggering Receptor Expressed on Myeloid Cells 2
T-tau	Total Tau
UCH-L1	Ubiquitin C-Terminal Hydrolase-L1
UPLC-Q-TOF-MS	Ultra-Performance Liquid Chromatography Coupled with Quadrupole Time-of-Flight Tandem Mass Spectrometry
VEGF	Vascular Endothelial Growth Factor
VEGFR-1	Vascular Endothelial Growth Factor Receptor
XGBoost	eXtreme Gradient Boosting
XPCT	Phase-Contrast X-Ray Imaging
WF-NTP	Wide Field-of-View Nematode Tracking Platform
YKL-40	Chitinase-3-Like Protein 1
3Rs	Replacement, Reduction, Refinement
